# Cambrian radiation speciation events driven by sea level and redoxcline changes on the Siberian Craton

**DOI:** 10.1126/sciadv.adh2558

**Published:** 2023-06-16

**Authors:** Andrey Yu. Zhuravlev, Rachel A. Wood, Fred T. Bowyer

**Affiliations:** ^1^ Department of Biological Evolution, Faculty of Biology, Lomonosov Moscow State University Leninskie Gory 1(12), Moscow 119234, Russia.; ^2^ School of GeoSciences, University of Edinburgh, James Hutton Road, Edinburgh EH9 3FE, UK.

## Abstract

The evolutionary processes of speciation during the Cambrian radiation and their potential extrinsic drivers, such as episodic oceanic oxygenation events, remain unconfirmed. High-resolution temporal and spatial distribution of reef-associated archaeocyath sponge species on the Siberian Craton during the early Cambrian [ca. 528 to 510 million years ago] shows that speciation was driven by increased endemism particularly ca. 521 million years (59.7% endemic species) and 514.5 million years (65.25% endemic species) ago. These mark rapid speciation events after dispersal of ancestors from the Aldan-Lena center of origin to other regions. These speciation events coincided with major sea-level lowstands, which we hypothesize were intervals when relative deepening of the shallow redoxcline permitted extensive oxygenation of shallow waters over the entire craton. This provided oxic corridors for dispersal and allowed the formation of new founder communities. Thus, shallow marine oxygen expansion driven by sea-level oscillations provides an evolutionary driver for sucessive speciation events during the Cambrian radiation.

## Teaser

Oxygen expansion driven by sea-level oscillations provides an evolutionary driver for speciation during the Cambrian Radiation.

## INTRODUCTION

The Ediacaran-Cambrian radiation marks the appearance and rise of diverse animals (metazoans) in the fossil record. In particular, the early Cambrian [ca. 538.8 to 510 million years (Ma) ago] saw the rapid emergence of all major phyla, complex metazoan reefs, modern-style food webs, as well as a substantial rise of metazoan abundance and biodiversity (*1*).

Biodiversity accumulates via speciation, and speciation by dispersal occurs when a population actively migrates into a new geographical area from the ancestral range. Regional diversification is promoted by either regional isolation or within-region environmental heterogeneity. Temporal and spatial habitat heterogeneity is known to be a major driver of biodiversification over evolutionary time scales (*2*). Modern marine invertebrate distributions are controlled mainly by varying dispersal ability in response to factors such as ocean currents, latitudinal/temperature gradients, and other physical barriers, rather than vicariance patterns, i.e., progressive continental fragmentation, usually by a physical barrier. Modern marine invertebrates can often disperse rapidly across large distances, via either propagules or using various rafting substrates, often driven by ocean currents (*3*).

Distinct biogeographic provinces were a feature of the lower Cambrian biota from its first appearance (*4*, *5*) [but see (*6*), for a different opinion]. Notable diversity hot spots fostering further differentiation of assemblages within provinces developed later [e.g., (*7*–*9*)]. Such a distribution may have been facilitated by the diverse types of metazoan propagules now reported from the lower Cambrian (*10*, *11*). On a global scale, between-assemblage diversity (β-diversity) increased rapidly in tropical regions during the Fortunian to Stage 3, together with a notable increase in provinciality, suggesting that it was regions dominated by single biostratigraphic zone species (singletons) that may have driven Cambrian provincialism, although no correlation was found between provinciality and diversity over the entire Cambrian (*12*). Tropical areas are also regions of preferential origin and persistence of species, from which species distributions later expand to higher latitudes (*13*). In the early Cambrian, higher latitude regions were represented by the continents of Avalonia and Baltica, with extremely low biodiversity and no endemics (*13*, *14*).

Four principal modes of speciation are recognized. Allopatric (or vicariant) speciation occurs when populations become isolated due to an extrinsic barrier that prevents gene flow. Peripatric speciation is promoted by similar isolating mechanisms that prevent gene exchange, but where new species form in an isolated peripheral population and one of the populations is much smaller than the other. In parapatric speciation, there are no specific, or only partial, extrinsic barriers to gene flow, but subpopulations evolve reproductive isolation in either continuous or discontinuous geographic ranges while continuing to exchange genes. Reproduction is nonrandom, and speciation occurs due to unequal or reduced gene flow within the population and varying selection pressures (*15*, *16*). Last, in sympatric speciation, both new and ancestral species continue to inhabit the same geographic region as no large-scale geographic distances are required to reduce gene flow between parts of a population. Here, exploitation of new ecological niches may reduce gene flow and cause speciation.

Now, we have no knowledge of the high-resolution dynamics during the Cambrian radiation that may reveal the operation of specific speciation processes within a single geographical province or metacommunity. To understand the potential extrinsic drivers of speciation events, integrated datasets are required that include temporal and spatial species distributions and their interrelationships, together with documentation of environmental change (*17*).

A causal relationship between shallow marine oxygenation and the Cambrian radiation has been proposed (*18*–*21*). The redox state of the Cambrian deep open ocean remains uncertain; however, early Cambrian seas likely had more shallow and dynamic redoxclines than that found in modern oceans, possibly reflecting the widespread maintenance of oxygen minimum zones (OMZs) along productive continental margins (*22*). Available data support the model that oxygenation during the Cambrian did not increase progressively and unidirectionally but rather proceeded via a series of short-lived oxygenation pulses, each of which lasted between 1 to 3 Ma, known as “oceanic oxygenation events” (OOEs) (*23*). These episodes of oxygen increase (potentially accompanied by productivity changes) are suggested to have expanded the area of oxygenated shallow-marine carbonate habitats on the Siberian Platform and hence to have promoted diversification (*20*, *21*). Synchronous and highly dynamic changes of the body size of hyoliths, helcionelloid molluscs, and archaeocyath sponges during the early Cambrian on the Siberian Craton coincide with elevated biodiversity and rates of origination, and these are hypothesized to have been driven by OOEs (*24*). Likewise, individual reef size and depth range of shelf occupation, as well as ecosystem complexity, also waxed and waned following proposed OOEs, also demonstrating that the Cambrian radiation progressed in an episodic, rather than a continuous linear way (*21*). The causal processes of diversification are not, however, established. While an increase in the extent of shallow habitable area alone may promote speciation, radiations have been further hypothesized to be driven by allopatric speciation where anoxic areas of shallow marine seawater created physical barriers to gene flow, creating heterogeneous habitats (*25*). Continued dynamic redox conditions would then have created successive bursts of radiation (*25*). Thus, the detailed dynamic pattern of the Cambrian radiation in relation to OOEs remains poorly quantified, as does insight into the evolutionary processes of speciation.

Here, we consider the temporal and spatial distribution of early Cambrian reef-building archaeocyath sponge species on the biodiversity hot spot of the Siberian Craton integrated with environmental data to provide insight into speciation dynamics and processes (*20*, *21*). We incorporate both outcrop and newly compiled subsurface borehole occurrence data within a revised age model (see Material and Methods, table S1, figs. S1 and S2, and Supplementary Data) (*26*). Reef communities are also loci of high biodiversity and, therefore, exert a major control on total marine diversity. A major metazoan turnover occurred after the extinction of the archaeocyath sponges during the Sinsk event toward the end of the early Cambrian (*12*, *27*), suggesting that the collapse of reefs further exerted a strong influence on overall biodiversity through the Cambrian radiation.

## RESULTS

### Geological setting and evolution of redox

**Fig. 1.**
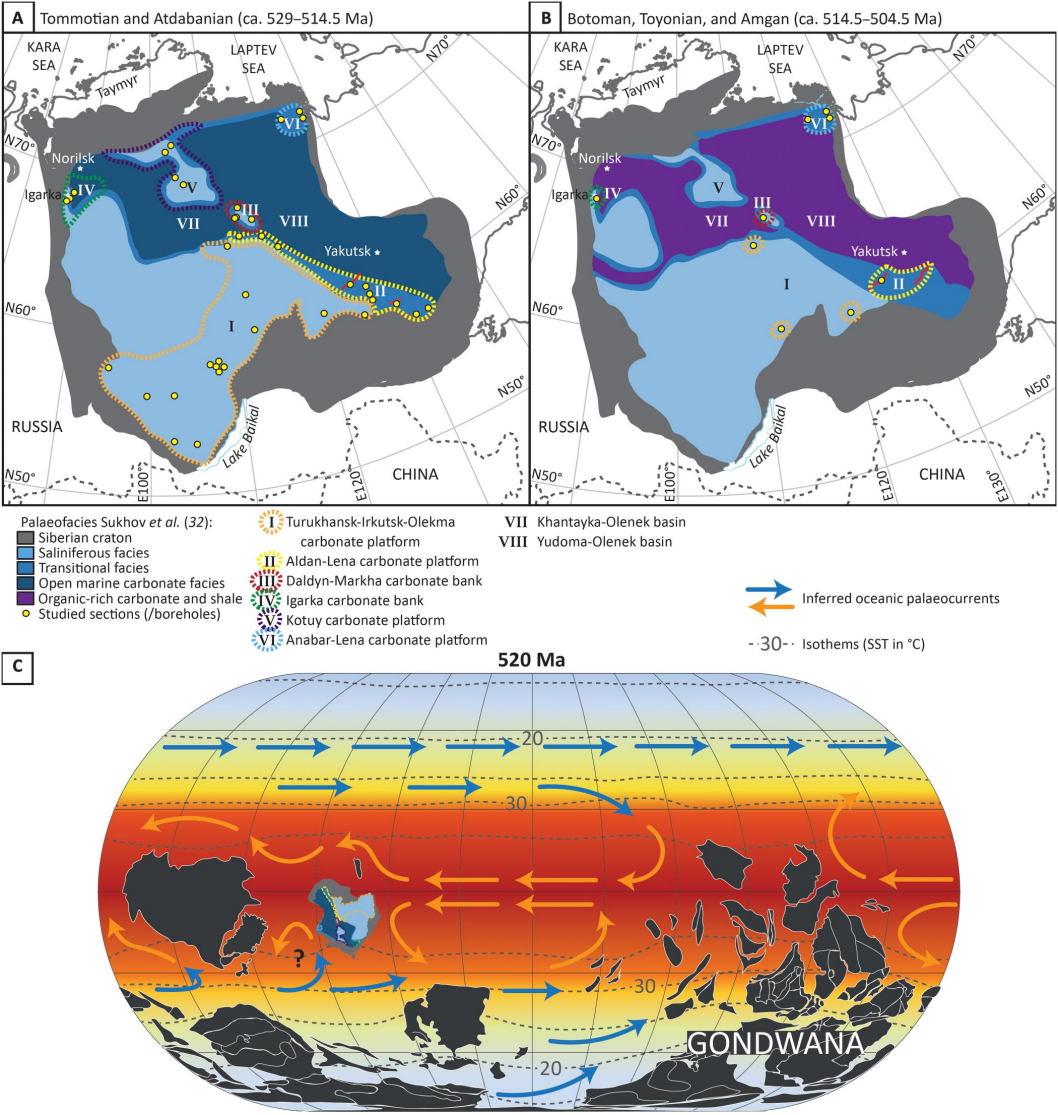
Paleofacies maps of the early Cambrian of the Siberian Craton, showing six areas (I to VI) with archaeocyath sponge occupation. (**A**) Tommotian to Atdabanian. (**B**) Botoman to Toyonian, and Amgan [modified from (*26*, *32*)]. (**C**) Cambrian paleogeography at 520 Ma ago after (*29*, *30*) with position of the Siberian Craton and possible ocean currents. Modeled sea surface temperature and isotherms after (*36*).

The Siberian Craton was a huge isolated, tropical continent during the early Cambrian, covered almost entirely by an epicontinental sea (Fig. 1), which, according to various paleomagnetic and plate tectonic models, was rotated approximately 180° relative to its present orientation (Fig. 1C) (*28*–*30*). This epeiric basin supported a single metacommunity of reef-building metazoans, i.e., a single species pool consisting of many local, interacting communities without immigration from elsewhere (*21*). The inner area of the Siberian Craton in the (present day) southwest area consisted of shallow marine carbonates together with thick evaporites and minor siliciclastics, known as the Turukhansk-Irkutsk-Olekma carbonate platform. Shallow marine marginal carbonates in the central area are known as the transitional facies zone (or the Anabar-Sinsk reef margin), which then passed to slope and deep ramp settings of the northern Khantayka-Olenek and eastern Yudoma-Olenek basins, which accumulated open marine carbonates during the Tommotian to Atdabanian (Fig. 1A) but organic-rich limestone and shale from the Botoman onward (Fig. 1B) (*31*, *32*).

During the Tommotian to Botoman stages, archaeocyaths inhabited six distinct shallow marine carbonate regions on the Siberian Craton, including the Aldan-Lena area on the Anabar-Sinsk reef margin, as well as the Igarka and Daldyn-Markha carbonate banks, and the Kotuy, Anabar-Lena, and Turukhansk-Irkutsk-Olekma carbonate platforms (Fig. 1, A and B; fig. S1; table S1; and Supplementary Data). These carbonate platforms and banks were mostly separated by the deeper Khantayka-Olenek and Yudoma-Olenek basins, with water depths too great for shallow reef building (*32*). Sedimentological analyses show that the Turukhansk-Irkutsk-Olekma carbonate platform was prone to elevated and variable salinity (Fig. 1, A and B) (*33*, *34*).

The general paleogeography of the Siberian Craton shows only minor changes through the early Cambrian, except for the separation of the Igarka bank from the Turukhansk-Irkutsk-Olekma carbonate platform during the Botoman and overall deepening of the Khantayka-Olenek and Yudoma-Olenek basins in the Botoman and early Toyonian (*32*) (Fig. 1B). Archaeocyath sponge reefs and bioherms were found mainly in the relatively shallow marine transitional facies. These reefs first appeared and expanded during the Cambrian stages 2 and 3 (Tommotian, Atdabanian, and earliest Botoman) but disappeared at the beginning of stage 4 (middle Botoman) during the Sinsk event extinction. Archaeocyaths reappeared briefly in two separate phases in the latest Botoman and middle Toyonian stages.

**Fig. 2.**
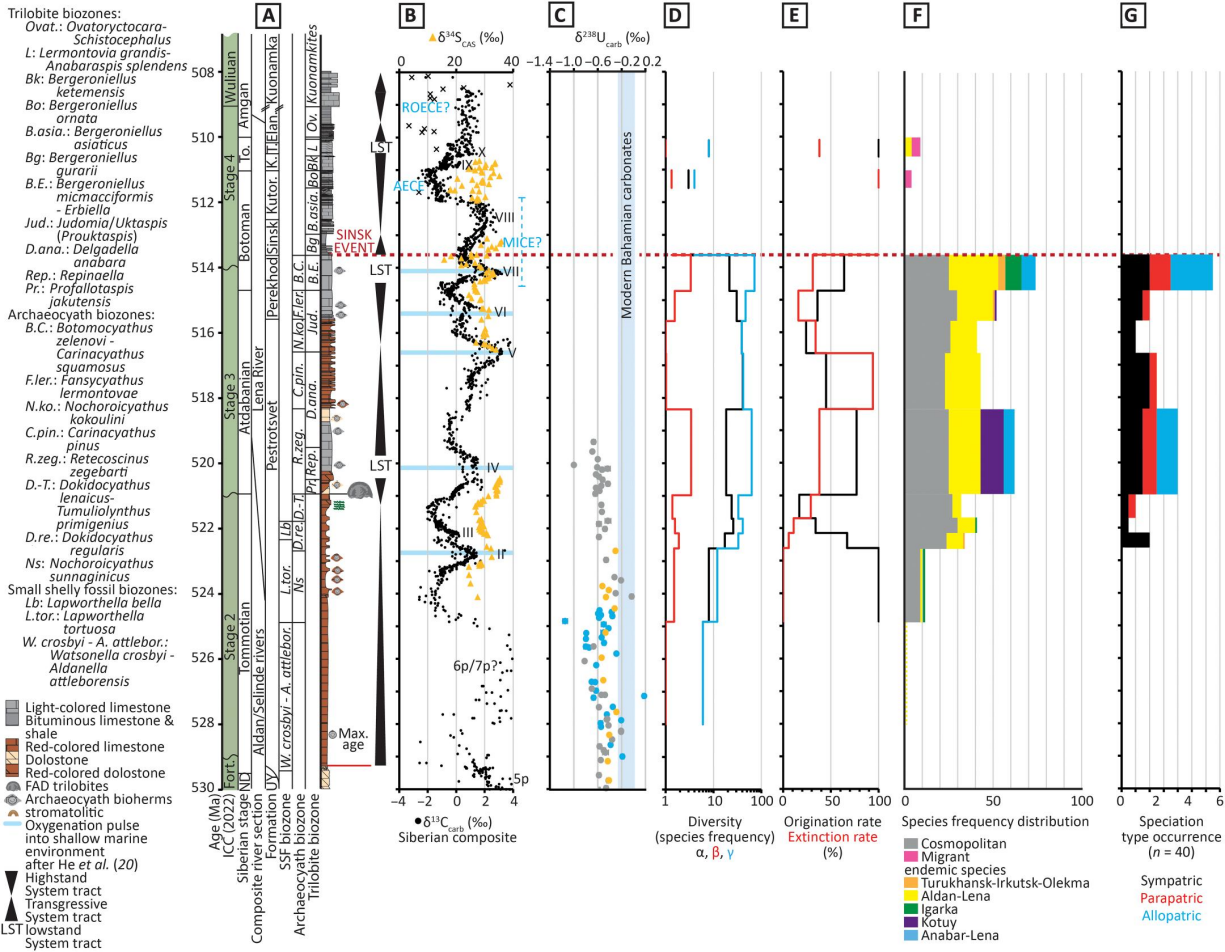
Summary of geochemical and biotic changes through the early Cambrian, Siberian Craton, and uranium isotope data representing a global record. (**A**) International (ICC) and Siberian time scale (*26*). Regional stages: Fortun., Fortunian; ND, Nemakit-Daldynian; To., Toyonian. Formations: U’-Y, Ust’-Yudoma; Ket., Keteme; T., Titary; Elan., Elanka. (**B**) Chemostratigraphic age model calibrating carbonate carbon isotopes [updated after (*26*)] (see Supplementary Data) and carbonate associated sulfate sulfur isotopes (*20*). (**C**) Uranium isotopes from Siberia (gray; Sukharikha and Bol’shaya Kuonamka rivers), South China (blue), and Morocco (orange) (all data points are larger than 2 SE) (*19*). (**D**) Archaeocyath sponge species α-, β-, and γ-diversity (*21*). (**E**) Rates of archaeocyath sponge species origination and extinction (*24*). (**F**) Distribution of cosmopolitan, endemic, and migrant species. (**G**) Distribution of speciation types (*n* = 40).

As far as now quantified, carbonate sedimentation on the Siberian Craton appears to have been deposited throughout the early Cambrian under consistently high, tropical temperatures (*35*, *36*). Sequence stratigraphic analysis recovers three incomplete third-order sequences, with sequence boundaries that coincide with Atdabanian 1 (ca. 520.5 Ma ago), Botoman 1 (ca. 514 Ma ago), and the mid-Toyonian (ca. 510.5 Ma ago) (Fig. 2A) (*20*, *21*, *26*).

During Cambrian stages 2 and 3, a strong positive covariation is shown between high-amplitude excursions of carbonate carbon isotopes (δ^13^C_carb_) and the sulfur isotope composition of carbonate-associated sulfate (δ^34^S_CAS_) in sections from the Aldan and Lena rivers (Fig. 2B) (*20*). The rising (positive) limbs of these contemporary excursions are suggested to mark phases of progressive burial of reductants within predominantly anoxic bottom waters, producing an increase in atmospheric oxygen. Coincident δ^13^C_carb_ and δ^34^S_CAS_ peaks (numbered II to VII) correspond with pulses of atmospheric oxygen into shallow marine settings, so producing an OOE. These were succeeded by a decrease in reductant burial under more widespread oxic conditions—the falling (negative) limbs of δ^13^C_carb_ and δ^34^S_CAS_—so leading to gradual deoxygenation over hundreds of thousands to millions of years (*20*). Within the age model framework refined here, the OOEs during δ^13^C_carb_ peaks IV, VI, and VII occur at ca. 520.5, 515.3, and 514.2 Ma ago, respectively (Fig. 2B).

Carbonate uranium isotope (δ^238^U_carb_) records are known from the Igarka bank (Sukharikha River), the Kotuy carbonate platform (Bol’shaya Kuonamka River), and the Yudoma-Olenek basin (Kotuykan River) of the Siberian Craton. These can be correlated with similar records in the Laolin and Xiaotan sections of South China and the Oued Sdas section of Morocco (*19*, *37*) and calibrated relative to the δ^13^C_carb_ record and archaeocyath biostratigraphy on the Siberian Craton only. These show a consistent pattern with δ^13^C_carb_ and δ^34^S_CAS_ (Fig. 2C), whereby decreasing values of δ^238^U_carb_ represent probably global expansions of anoxic (and likely euxinic) conditions, that were conducive to more widespread burial of reductants (*38*, *39*). Values of δ^238^U_carb_ reach a nadir during δ^13^C_carb_ peak 6p but recover to more positive mean values during the late Cambrian stage 2 to early stage 3, which may record a slow transition to a less euxinic or reducing deep ocean globally, characterized by continued redox stratification and/or a reduction in the spatial extent of organic carbon and pyrite burial under widespread OMZ-type conditions (*40*, *41*).

### Archaeocyath species distribution

Archaeocyaths first appear as fragmentary material where species cannot be determined in the lowermost Pestrotsvet Formation on the Selinde River (Aldan-Lena area) during δ^13^C_carb_ peak 6p/7p below the base of the *Nochoroicyathus sunnaginicus* Zone and, therefore, have a maximum age coincident with the lowermost Tommotian (*26*, *42*). Previous reports (*43*) of the occurrence of archaeocyaths in the topmost Sukharikha Formation on the Sukharikha River (Igarka bank) have proved unsubstantiated (see Stratigraphic Notes).

From the initial appearance of archaeocyaths, their γ-diversity (total raw species diversity; *n* = 205) on the Siberian Craton increased progressively to 62 species by the early Atdabanian *Retecoscinus zegebarti* Zone (Fig. 2D). After a decline during middle Atdabanian, species diversity increased during the late Atdabanian to reach 72 species by the early Botoman. A coverage-standardized curve shows that, once sampling is taken into account, this general pattern of diversity changes is essentially stable (*21*).

Origination rates of archaeocyaths also similarly increased in two steps, during the early Atdabanian and the late Atdabanian to early Botoman (Fig. 2E) (*24*). The middle Atdabanian and middle Botoman show peaks in archaeocyath extinction rates, where the middle Botoman peak marks the Sinsk extinction event that caused in the near extinction of the archaeocyaths and many other calcified metazoans (*27*). α-Diversity reaches a modest peak in the mid-late Tommotian and a more pronounced peak in the mid-late Atdabanian. β-Diversity reached peaks in the middle Tommotian, early Atdabanian, and early Botoman (Fig. 2D and Supplementary Data). New species of archaeocyaths appear briefly in the late Botoman and middle Toyonian and, thereafter, become extinct on the Siberian Craton.

**Fig. 3.**
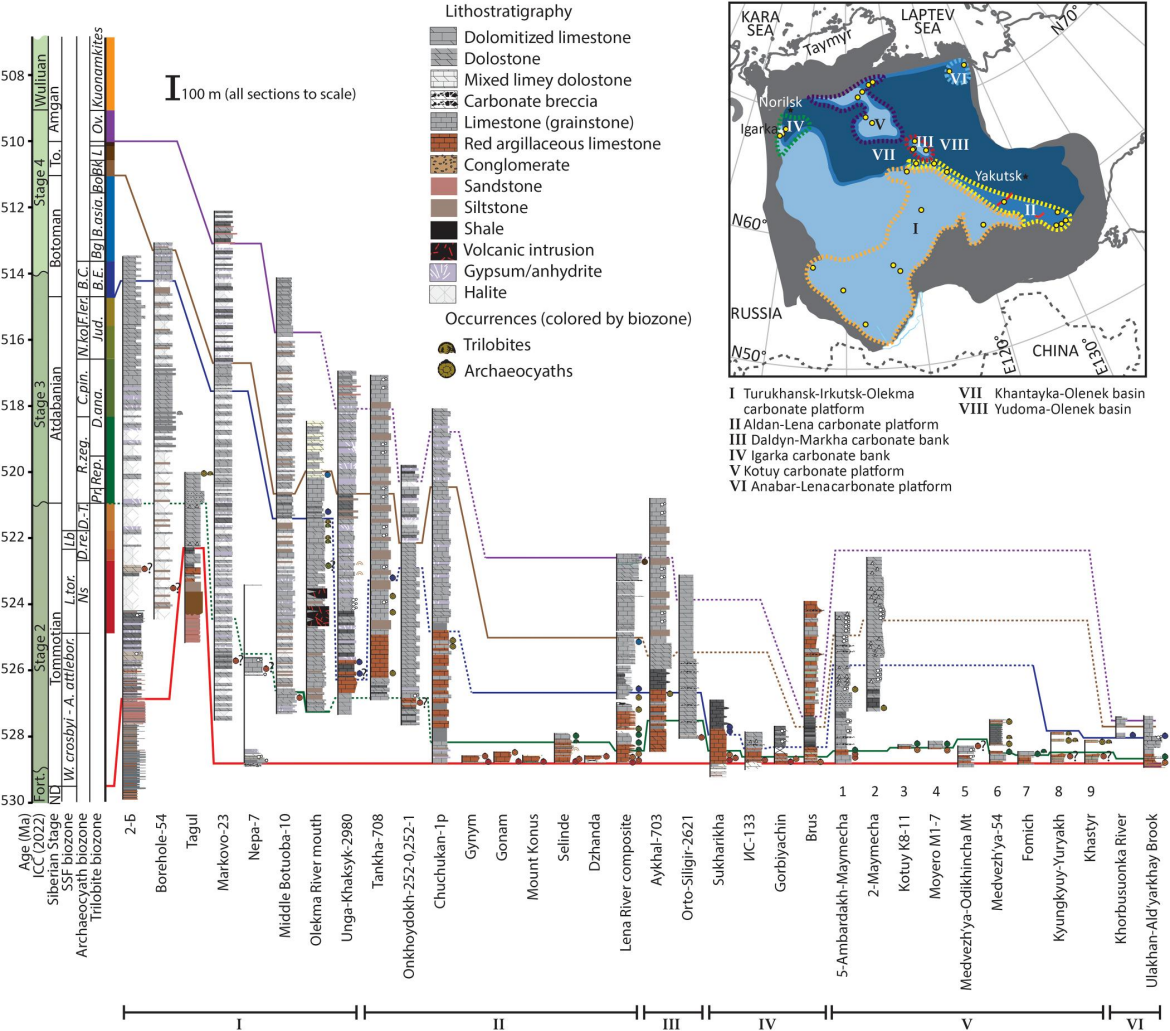
Summary of correlation framework for boreholes/sections of the Siberian Craton. Full correlation and references provided in fig. S1, full version, and the Supplementary Materials.

**Fig. 4.**
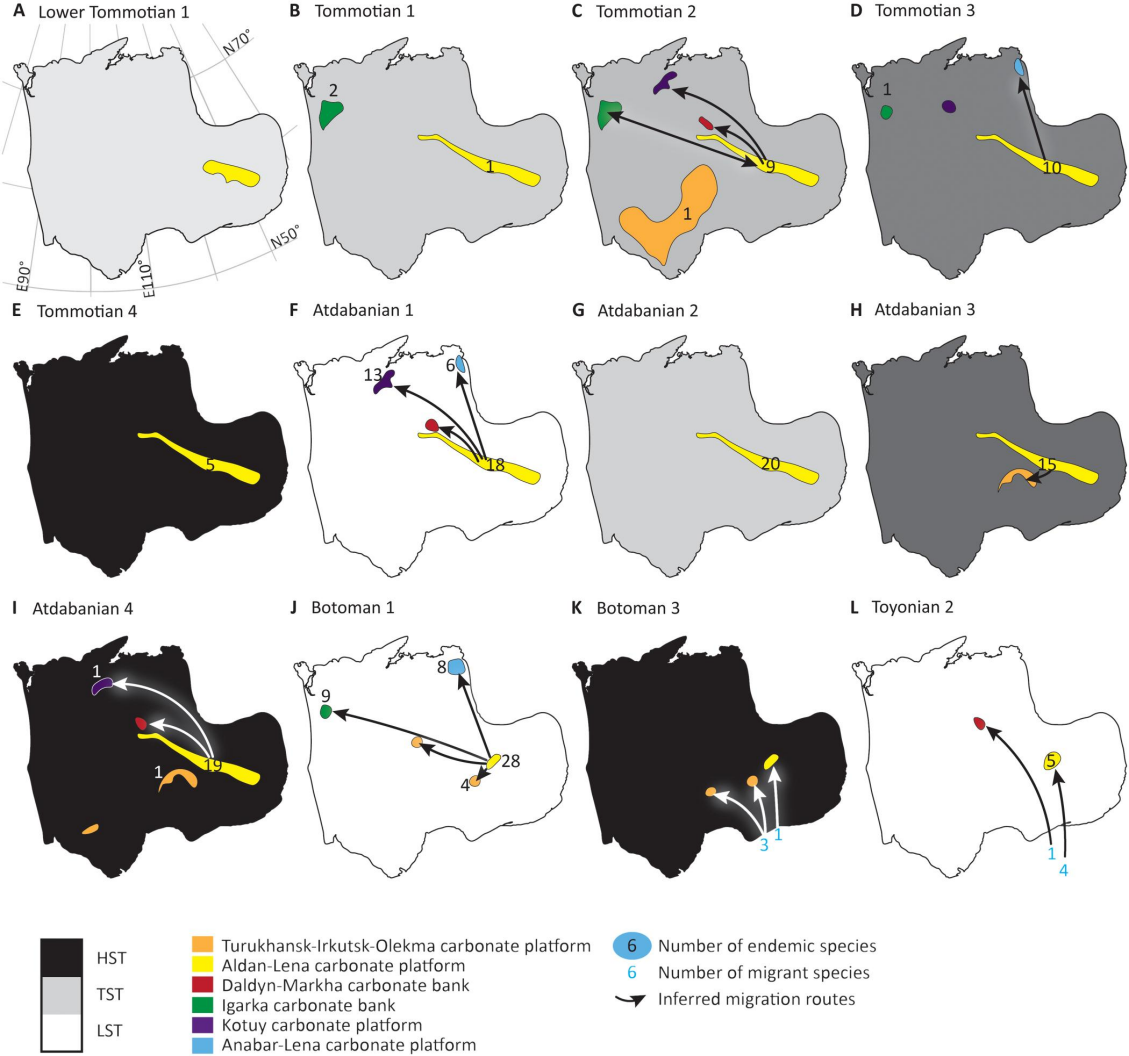
Distribution of archaeocyath sponge species on the Siberian Craton. (**A** to **L**) Time intervals showing regions of occupation and loss, endemic species distribution, and inferred migration pathways. These are integrated with the distribution of other metazoan benthos and relative sea-level change, shown by gray shading. HST, highstand system tract; TST, transgressive system tract; LST, lowstand systems tract.

In total, there are more endemic (*n* = 120, 58.6%) than cosmopolitan (*n* = 78, 38%) species (Supplementary Data). The Aldan-Lena area was continuously inhabited by archaeocyaths from the earliest Tommotian to earliest Botoman and yields the largest number of both endemic and cosmopolitan species from the Tommotian 2 onward. All other regions have distinct temporal records of endemism: The Kotuy carbonate platform was colonized only during the Tommotian 2 and 3 and Atdabanian 1 and 4; the Igarka bank during the Tommotian 1 to 3 and Botoman 1; the Turukhansk-Irkutsk-Olekma carbonate platform during the Tommotian 2, Atdabanian 3 and 4, and Botoman 1 and 3; the Anabar-Lena carbonate platform during the Tommotian 3, Atdabanian 1, and Botoman 1; and the Daldyn-Markha carbonate platform during the Tommotian 2, Atdabanian 1 and 4, and Toyonian 2 (Figs. 2F, 3, and 4; figs. S1 and S2; table S1; Supplementary Data).

During the Botoman 3 and Toyonian 2, we note the first probable migrant species (*n* = 6) from outwith the Siberian Craton (Fig. 2F). These are new species without any close relatives on the Siberian Craton and include representatives of the genera *Irinaecyathus*, *Kiwicyathus*, *Erbocyathus*, *Tegerocyathus*, *Cellicyathus*, *Claruscoscinus*, and *Archaeocyathus*, most of which were not present on the Siberian Craton previously but were widespread in the Botoman and Toyonian reefs of the Altay-Sayan and Mongolian terranes (*44*, *45*). *Irinaecyathus* and *Erbocyathus* appear only in the Botoman 1 and 3, respectively, but a gap in their stratigraphic distribution together with *Erbocyathus heterovallum* (again typical of the Altay-Sayan Foldbelt) supports their collective origin beyond the Siberian Craton. All other Botoman 3 and Toyonian 2 endemic species (*n* = 5) on the Siberian Craton that belong to the same genera are their direct descendents.

Apart from the initial appearance of the group, the proportion of endemic species follows diversity, with two marked peaks of endemism in the Atdabanian 1 (59.7% species) and Botoman 1 (65.27% species) (Fig. 2F). In the Botoman 1, endemic species are known in 34 genera, and almost a third of these genera are endemics represented by a single species each (table S1).

Many endemic species are singletons (74%), and only such singletons are present in the Anabar-Lena, Kotuy, and Turukhansk-Irkutsk-Olekma carbonate platforms and on the Igarka bank. Cosmopolitan species are notably longer-lived (mean, 5.37 Ma; *n* = 78) than endemic species (mean, 1.48 Ma; *n* = 120).

### Phylogenetic relationships

Archaeocyath sponge species are recognized by shared morphological and ontogenetic development and obligate ecological niches (*45*–*47*). Extant sponges show a close association between morphological and species-specific genetic traits (*48*–*50*).

The phylogeny of the Archaeocyatha is not, however, established because of a scarcity of diverse morphological features, which can also recur in unrelated lineages (*51*), but individual ancestor-descendant pairs can be deduced for some species (Supplementary Data). Such presumed phylogenetic relationships are determined by (i) shared skeletal features, with only minor variations; (ii) early stages in the skeletal ontogeny of the descendent species that share the morphology of the ancestral species; (iii) an absence of any other morphologically close potential relatives on the Siberian Craton, and (iv) their successional appearance in any given locality, sometimes with a slight overlap of their stratigraphic ranges (Fig. 3 and fig. S1).

For instance, in the succession *Tumuliolynthus primigenius–Tumuliolynthus* “*tubexternus*”–*Tumuliolynthus karakolensis*, each younger species has successively larger pores and tumuli (*46*, *52*, *53*). *Erismacoscinus fedorovi*, *Erismacoscinus oymuranensis*, and *Erismacoscinus batchykensis* probably represent a single lineage, where each successive species has a slightly higher number of pore rows per intersept in both cup walls. *Retecoscinus sakhaensis* has a number of features in common with its stratigraphic predecessor *Erismacoscinus rojkovi*, differing only by the porosity of the tabulae, and in the lineage *Coscinocyathus isointervallumus*– *Coscinocyathus marocanoides*; the latter has larger inner wall bracts (*54*).

Calibration and correlation of regional sections by δ^13^C_carb_ data for the Aldan-Lena area, Igarka bank, and Anabar-Lena platform (*42*, *55*–*57*) in addition to other regions (*26*) allow determination of the place of origin of species and potential routes of migration. For instance, the early Botoman archaeocyath *Carinacyathus squamosus–Botomocyathus zelenovi* Zone, in general, covers the interval that records the δ^13^C_carb_ positive peak VII, but the species characterizing this zone appear at the beginning of this positive excursion in the Aldan-Lena area, during the excursion on the Anabar-Lena platform, and at the acme of this positive excursion on the Igarka bank. First appearance and duration of ancestor-descendant species, as well as their geographic distribution, can thus be established. From this, the evolutionary origin of some cosmopolitan and endemic species can be determined and, in turn, used to infer regional relationships (see fig. S1).

Using such reasoning, 41 such ancestor-descendent pairs have been identified (Supplementary Data). For example, the early Tommotian *Archaeolynthus polaris1* gave rise to the late Tommotian *T. primigenius*, which, in turn, was ancestral for the Atdabanian to early Botoman *T. tubexternus* and *T. karakolensis*. In the Atdabanian, *Carinacyathus kigitasensis* gave rise to the cosmopolitan late Atdabanian *Carinacyathus pinus* and early Botoman *C. squamosus*. In the middle Atdabanian, *Tumulocoscinus botomaensis* evolved to the cosmopolitan late Atdabanian to early Botoman *Tumulocoscinus atdabanensis*.

Here, for simplicity, we accept that archaeocyath species pairs are allopatric if they either coexisted or succeeded each other temporally in different regions (fig. S1 and Supplementary Data). In a strict sense, this may mainly represent peripatric speciation where a larger ancestral population produces smaller and geographically distinct founder populations (*16*). We are not, however, able to estimate founder population sizes in quantitative terms because archaeocyath reef communities do not represent initial settlements. Species that co-occur within the same reef communities (outcrops or boreholes), i.e., are represented by the same sampling set from the same region, are cautiously interpreted here as “sympatric.” Sympatric speciation is not easily detected even in modern ecosystems, but, here, this potential mode should be distinguished from that where related species are restricted to the same region (from the same or different localities) but replace each other temporally, i.e., are always present in stratigraphically different reefs communities. Here, we tentatively recognize this phenomenon as parapatric speciation (see Supplementary Data).

Where mode can be identified in ancestor-descendent pairs, 52.5%, 25%, and 22.5% represent sympatric (or potential peripatric), parapatric, and allopatric speciation, respectively (*n* = 40). Parapatry and “sympatry” are noted only in the Aldan-Lena region, and allopatry in the Igarka, Kotuy, Anabar-Lena, and Turukhansk-Irkutsk-Olekma regions. Sympatry is present during the entire interval, parapatry during the late Tommotian to mid Atdabanian and the late Atdabanian to early Botoman, but allopatry is only identified during the early Atdabanian and early Botoman events (Fig. 2G and Supplementary Data).

All ancestral species of those deduced to be related are either cosmopolitan or endemic to the Aldan-Lena area. Cosmopolitan-to-cosmopolitan ancestor-descendent relationships are the most frequent (*n* = 21, 52.5%). Some endemic species gave rise to cosmopolitan species (*n* = 4, 10%), e.g., *Sibirecyathus onkhoydokh* (endemic to the Aldan-Lena area) fostered *Sibirecyathus suvorovae*, which then spread to almost all other areas of the Siberian Craton. Cosmopolitan species also gave rise to endemic species (*n* = 10, 25%), e.g., *Tumulocyathus kotuyikensis* to *Tumulocyathus danieli* (endemic to Kotuy). Last, there are occurrences of endemic to endemic species relationships (*n* = 5, 12.5%), e.g., *Taylorcyathus taylori*, which originated on the Aldan-Lena and then gave rise to *Taylorcyathus milashevae* in the Kotuy region.

### Benthic metazoan distribution

Not all regional carbonate banks and platforms show the continuous occupation of a benthic metazoan biota (Fig. 4). The Daldyn-Markha bank in particular shows a very episodic history, now recorded only in boreholes. Small shelly fossils (SSFs) and archaeocyaths are recorded during the Tommotian 2, but abundant archaeocyaths, spiculate sponges, SSFs including molluscs, some trilobites, and linguliformean brachiopods are present during the Atdabanian 4, and trilobites, brachiopods, and paleoscolecidans have been recorded during the Toyonian 3 to Amgan interval (*32*, *58*).

On the Igarka carbonate bank, rare trilobites, hyoliths, and linguliformean brachiopods, but no SSFs are present during the Atdabanian 2 to 4 and Botoman 2 and 3. Abundant trilobites and brachiopods briefly reappeared in the Toyonian 2 (*43*, *59*).

A similar pattern of biotic distribution is observed on the Kotuy and Anabar-Lena carbonate platforms, with SSFs occurring intermittently during the Nemakit-Daldynian, Tommotian 2 and 3, Atdabanian 1 and 4, Botoman 1, Toyonian 2, and Amgan intervals (*43*, *57*, *60*–*62*).

## DISCUSSION

Archaeocyath species distribution is spatially very dynamic on the Siberian Craton, with variable occupation of the six regions (Figs. 2F and 4, figs. S1 and S2, and Supplementary Data). Regions episodically developed endemic biotas, with the largest, the Aldan-Lena, remaining as the main center of species diversification and origination (Fig. 4). This was the only area of continuous Tommotian 1 to Botoman 1 archaeocyath speciation (Figs. 2F and 4). The Botoman 1 marks the peak of archaeocyath species diversity (Fig. 2D). This peak coincides with the peak of all known skeletal metazoan species diversity on the Siberian Craton (*24*), suggesting that the speciation processes for archaeocyath sponges might also be in operation for other groups.

Speciation (as shown by high rates of origination) maintains diversity and is driven by an increase in the number of endemic rather than cosmopolitan species during two intervals: ca. 521 Ma ago (the early Atdabanian) with 59.7% endemic species marking speciation events in the Aldan-Lena, Kotuy, and Anabar-Lena, all regions except the Daldyn-Markha carbonate platform (Fig. 4F); and ca. 514.5 Ma ago (the early Botoman) with 65.27% endemic species (Supplementary Data). During Botoman 1, speciation events occurred in all regions except the Daldyn-Markha and Kotuy carbonate platforms (Fig. 4J). These correspond to peaks in β-diversity, confirming an increase in interregional differences. A minor diversification event also occurred in the Igarka, Kotuy, Anabar-Lena, and Aldan-Lena areas during Tommotian 2 (middle stage 2; ca. 525 Ma ago) (Figs. 2, D and F, and 4C). Speciation events occur in all regions except the Daldyn-Markha carbonate bank.

In summary, endemic species are notably shorter-lived than cosmopolitan species (mean, 1.48 versus 5.37 Ma, respectively), and most are singletons. Endemic versus cosmopolitan species are unlikely to show any differences in propagule dispersal capabilities or longevity as they are often members of the same genera (Supplementary Data). The short-lived nature of endemic species is perhaps due to the fact that broadly distributed species due to a higher diversity and wider distribution of populations are more resistant to extinction than those with narrow ranges (*63*–*65*).

The increased ability of propagules to disperse does not show any directional temporal trends. It is possible, however, that the paleogeographic distribution of species may not be dependent on dispersal ability but rather on apparent taxonomic duration (*66*). Many Cambrian groups may have been taxonomically oversplit, including archaeocyath sponges (*45*), SSFs (*67*), and trilobites (*68*). Among archaeocyaths, for example, of almost 2000 species described, about 550 (>25%) are either synonyms or nomina dubia. There is no reason to believe, however, that certain time intervals during the early Cambrian will contain disproportionally more oversplit species, and so the two phases of increased endemicity described here are likely to be real.

The intervals ca. 521 and 514.5 Ma ago mark major speciation events, with examples of allopatric, parapatric, and sympatric (or potential peripatric) speciation (Fig. 2G). In those ancestor-descendant relationships discerned (*n* = 40; Supplementary Data), sympatry is the dominant mode of speciation, and parapatric and allopatric speciation are broadly equally represented. Parapatry and sympatry are noted only in the Aldan-Lena region, and allopatry only in Igarka, Kotuy, Anabar-Lena, and Turukhansk-Irkutsk-Olekma regions. Minor probable sympatric speciation events are present during the Tommotian 1 and 3 on the Igarka bank. Sympatry and parapatry were found in the Tommotian, late Tommotian to early Atdabanian, and late Atdabanian to early Botoman speciation phases, but allopatry was only found in the latter two and most notable speciation events (Fig. 2G).

These data are used to infer the dispersal routes of cosmopolitan species and ancestors of future endemic species from the Aldan-Lena area to other regions during the Tommotian 2 (to Kotuy, Daldyn-Markha, and Igarka; Fig. 4C and Supplementary Data), the Atdabanian 1 (to Kotuy, Anabar-Lena, Dalyn-Markha; Fig. 4F), and the Botoman 1 (to Anabar-Lena, Igarka, and Turukhansk-Irkutsk-Olekma; Fig. 4J). The Daldyn-Markha carbonate platform was also colonized from the Aldan-Lena area during the Tommotian 2 and Atdabanian 1 and 4 (Fig. 4I).

Dispersal alone did not create an increase in biodiversity. If reconstructed paleolatitudes are correct, then the Aldan-Lena area was in the northwestern corner of the Siberian Craton, and so the principle ocean current direction should have dispersed the propagules toward Laurentia, rather than into the Siberian Plate interior as described here (Fig. 1C). Any paleocurrents running in a westerly direction, however, would meet the shallow water Anabar-Sinsk barrier and so might deviate, at least partially, toward the South-East. All ancestors of those species discerned to be related are either cosmopolitan or endemic to the Aldan-Lena. Migration from the Aldan-Lena area, therefore, episodically extended cosmopolitan and endemic species ranges into new regions and facilitated allopatric and parapatric speciation. Of these, cosmopolitan-to-cosmopolitan ancestor-descendent relationships are the most frequent, but endemic species can also give rise to cosmopolitan species, and cosmopolitan species can give rise to endemic species.

The Sinsk event marked an extinction that removed all archaeocyaths from the entire Siberian Craton. Archaeocyaths returned during the Botoman 3 due to the migration of new species to the Aldan-Lena and the Turukkhansk-Irkutsk-Olemka platforms from the Altay-Sayan and Mongolian regions. A protracted sequence of tectonic processes in the Altay-Sayan and Mongolian regions caused the accretion and amalgamation of these regions to the Siberian Craton in the late Botoman-Toyonian (*28*, *69*), which may have facilitated migration from these regions when local species on the Siberian Craton had already become extinct (Fig. 4, K and L). Settlement of these migrants was restricted to the present southeastern Siberian Craton only, the region closest to the accretionary belt. During the Toyonian 2, migrant species of *Irinaecyathus*, *Tegerocyathus*, and *Archaeocyathus* gave rise to new endemic species descendants via allopatric speciation.

All six regions show available shallow marine carbonate platform habitats throughout the early Cambrian (Fig. 1). We note an absence of the formation of any tectonic barriers, which might have induced vicariance. Most regions supported at least an episodic metazoan benthos throughout the early Cambrian interval (*70*, *71*), and we infer that these shallow marine carbonate platforms were well-oxygenated when a metazoan benthos was present. However, hyoliths, molluscs, and SSFs almost disappeared from the Igarka bank as wells as Kotuy and Anabar-Lena carbonate platforms during the Atdabanian 2 and 3 and Botoman 2 and 3 (Fig. 4). Like archaeocyath sponges, these groups had calcareous skeletons (and tommotiids had robust phosphatic sclerites), so it is possible that they were less buffered to redox changes or lower oxygen levels (*24*).

The absence of benthos on the Daldyn-Markha bank, adjacent to the Aldan-Lena area, however, may indicate persistent reducing or anoxic conditions except for brief episodes during the Tommotian 2, Atdabanian 1 and 4, and Toyonian 2. An even more restricted benthos typifies the Turukhansk-Irkutsk-Olekma platform, represented by a limited number of archaeocyaths, trilobites, and a few other taxa. This vast area is characterized by thick lower Cambrian evaporites represented by the Usol’e, Bel’sk, and Angara Formations indicative of sustained, elevated salinity (*34*): In the borehole-54 of the Cis-Sayan area, the thickness of the Usol’e Formation alone reaches 2300 m (Fig. 3) (*33*). Except for a brief episode during the Tommotian 2, when ca. seven species of very morphologically simple archaeocyaths associated with calcimicrobial reefs became widespread over this platform (*72*), archaeocyaths were restricted to a narrow zone abutting the Anabar-Sinsk reef margin during only the Atdabanian 3 to Botoman 1 interval (Fig. 4, F, L, and J). The last archaeocyaths on the Turukhansk-Irkutsk-Olekma platform appeared in the Toyonian 2, and these were migrants restricted to a few areas on the southeast part of this area (Fig. 4L). With the exception of the Turukhansk-Irkutsk-Olekma platform, the switch from open marine carbonates in deep ramp and slope settings during the Tommotian to Atdabanian to organic-rich limestone and shale from the Botoman 2 onward suggests a deepening of the basin but may also indicate a shallowing of the redoxcline during this interval, conducive to the enhanced preservation of organic matter under reducing bottom waters.

Under the low-oxygen conditions inferred for the early Paleozoic atmosphere and ocean, sea-level rise may have passively induced an upward movement of the redoxcline, leading to widespread anoxic deposition across the Siberian Platform. In addition, during the lower Cambrian, the low latitude position and inverted orientation of the Siberian Craton would have placed the Yudoma-Olenek basin in proximity to a region of the open ocean that may have been inpinged by the upwelling of nutrient rich deep water (Fig. 1C). Intervals of sea-level rise may, therefore, have promoted nutrient incursion from the open ocean, so stimulating enhanced primary productivity in surface waters of the Siberian Craton and resulting in OMZ expansion, organic carbon remineralization, and active shallowing of the redoxcline.

We propose that allopatric speciation in archaeocyaths was related to their dispersal from a center of origin (the Aldan-Lena area) to other carbonate platforms and banks when these became oxygenated. The two episodes of high diversity that coincide with elevated archaeocyath speciation during the Atdabanian 1 and Botoman 1 and the limited endemic speciation of archaeocyaths in the Toyonian 2 notably coincide with third-order relative sea-level lowstands (Figs. 2A, and 4, F and J). The apparent record of fossil species distribution spatially across a shelf can be controlled by hiati, nondeposition, and changes in relative water depth (*73*). However such a concentration of biodiversity coincident with lowstands is unlikely to be a sampling artefact, as stratigraphic models have shown that first and last appearances of taxa are rather clustered preferentially at sequence boundaries and at major flooding surfaces (*74*). We hypothesize that intervals of low relative sea level shifted the position of the inferred shallow redoxcline off the shallow carbonate platform. This potentially created oxic corridors for dispersal and allowed new founder communities to form via allopatric speciation. The Daldyn-Markha bank, for instance, can be inferred to have become temporarily oxygenated only during the Tommotian 2 and Atdabanian 1 and 4, the latter of which, although coinciding with a sea-level highstand (Fig. 4I), occurred when reef facies (dendrolites and variegated argillaceous mud mounds surrounded by grainstone) indicate that water depths over this area were very shallow (*32*, *58*). By contrast, the intervening transgressive middle Atdabanian interval (ca. 519 to 516 Ma ago) corresponds not only to a marked decline in regional occupation, endemicity, and a decrease in total species diversity driven by increased rates of archaeocyath extinction but also to the loss of other heavily calcified biota (hyoliths, molluscs, and calcareous SSFs) on the Igarka bank and Kotuy and Anabar-Lena carbonate platforms (Figs. 2, D and E, and 4, G and H). Progressive expansion of low-oxygen waters into shallow marine platform areas during transgression may have created local extinction. The Sinsk extinction during Botoman 2 may have been driven by a more profound and widespread increase in shallow marine reducing or anoxic conditions indicated by widespread accumulation of organic-rich facies (*75*), among which hopanes of sulfate-reducing, methanotrophic, and methylotrophic prokaryotes are common (*76*) and decoupled δ^13^C_carb_ and δ^34^S_CAS_ records (*20*), resulting in the virtual demise of archaeocyath sponges and much of the associated reef biota (*27*).

Sympatric and parapatric speciation in archaeocyaths on the Siberian Craton may have been driven by high environmental heterogeneity, perhaps also promoted by the variable oxic habitat of shallow water carbonate areas that fluctuated due to frequent redoxcline oscillations. Niche partitioning due to a dietary specialization may also have been a factor. Archaeocyaths have highly variable skeletal elements that represent fine sieves and other devices that enhance water filtration due to the Bernoulli effect; Their entire evolution may have been governed by the rapid and continuous refinement of these features (*77*, *78*). Archaeocyath species are also noted to segregate into several obligate ecological niches adapted to various substrates and microenvironmental conditions (*60*). Complex topographic structures, such as found on reefs, can facilitate speciation even more than complete spatial isolation (*79*). Such differentiation might have produced isolated populations similar to those observed in extant freshwater fish [for a summary, see (*80*)]. Sympatric speciation may have been further promoted by disruptive selection due to competition for a limited uni- or bimodally distributed current-borne food resources (*81*, *82*). Archaeocyath species adaptations via morphological changes may have affected their success in dispersal of propagules, so further promoting reproductive isolation. Thus, the principal trends of archaeocyath evolution that led to differentiation of their eco-morphological traits and resource utilization may have enhanced sympatric speciation.

In conclusion, analysis of the speciation of reef-building archaeocyath aspiculate-hypercalcified sponges on the Siberian Craton during the early Cambrian stages 2 and 3 (ca. 528 to 510 Ma ago) indicates that this group diversified episodically via sympatric, parapatric, and allopatric modes within six regional shallow marine carbonate banks and platforms.

Of these six regions, only the Aldan-Lena area of the Anabar-Sinsk reef margin remained a continuous center of the species origination and diversification throughout the early Cambrian. Speciation was driven here and, in other regions, by increased endemism particularly during two intervals: early Cambrian stage 3, ca. 521 Ma ago (early Atdabanian; 59.7% endemic species), and late stage 3, ca. 514.5 Ma ago (early Botoman; 65.25% endemic species).

Sympatric and parapatric speciation in archaeocyaths on the Siberian Craton may have been driven by high environmental heterogeneity due to frequent redoxcline oscillations and differentiation of their eco-morphological traits and resource utilization. Dispersal alone did not create an increase in biodiversity, but these major speciation events coincided with major sea-level lowstands, which we hypothesize were intervals when relative deepening of the shallow redoxcline permitted extensive oxygenation of shallow marine waters over the entire craton. This supports the hypothesis that radiations can be driven by allopatric speciation where anoxia creates physical barriers to gene flow, and continued redox oscillations can promote successive bursts of radiation (*25*). Hence, we demonstrate that the highly dynamic history of shallow marine oxygen distribution driven by sea level provides a mechanistic evolutionary driver for speciation during the Cambrian radiation.

## MATERIALS AND METHODS

We document the temporal and spatial distribution of 205 species of reef-associated archaeocyath sponges on the Siberian Craton during their entire Early Cambrian record to quantify the timing of potential sympatric and allopatric speciation events and, therefore, to test the evolutionary response to OOEs and other extrinsic drivers. We consider data from 25 archaeocyath-bearing outcrops and boreholes from six paleogeographic regions established in (*32*) (Figs. 1 to 3, fig. S1, table S1, and Supplementary Data), namely, the Aldan-Lena area (incorporated in the southeastern Anabar-Sinsk reef margin) as well as the Igarka (the Igarka-Noril’sk Uplift) and Daldyn-Markha banks; and Kotuy (the Anabar Shield western slope), Anabar-Lena (including the Khara-Ulakh Mountains and Olenek Uplift), and Turukhansk-Irkutsk-Olekma carbonate platforms.

Species occurrences with the qualifiers “cf.,” “aff.,” “n.sp.,” or “indet.” were disregarded except for extremely rare finds from the inhospitable Turukhansk-Irkutsk-Olekma carbonate platform, which represent distinct morphs. The final dataset comprises 53 collections from the Aldan-Lena area and 94 collections from other areas constituting 205 archaeocyath species in total (see table S1, Supplementary Data, and references). The identification of species made by different authors of the aforementioned publications has been checked from thin sections housed in the Borissiak Palaeontological Institute (Moscow); Central Siberian Geological Museum of Institute of Geology and Mineralogy of the Siberian Branch (Novosibirsk); and Russian Academy of Sciences, and Siberian Scientific-Research Institute of Geology, Gephysics and Mineral Resources (Novosibirsk). Species were identified and counted in thin sections, and species co-occurrences were also identified at the scale of individual thin sections. Between 10 and 50 thin sections were analyzed per locality depending on thin section area (2 to 400 cm^2^), which were then collated.

The temporal and spatial distribution of archaeocyath species is used to derive γ-diversity (total species richness across all aforementioned areas), α-diversity (mean species richness within regions, strongly controlled by local ecology and niche requirements of species), and β-diversity (γ-diversity divided by mean α-diversity, which indicates the differences between regions). Archaeocyath species rates of origination and extinction were assembled from (*24*). Extinction rate is the number of taxa that did not extend into an interval divided by total species diversity (γ-diversity), and origination rate is the number new taxa that appear in that interval divided by the total diversity. These macroevolutionary rates presented are regional rates such that speciation rate is a combination of true speciation and immigration, and extinction rates are a combination of true extinctions and extirpations.

We also quantify the distribution of endemics restricted to a single area versus cosmopolitan species, which occurred in two or more regions, and calculated species’ stratigraphic durations by the number of stratigraphic intervals over a species are found. Appearances of migrants from beyond the Siberian Craton are also considered. The phylogeny of archaeocyath sponges is not established, but genera and species are well defined. Ancestor-descendent pairs were identified (*n* = 41), and these were used to determine styles of speciation (Supplementary Data).

No local redox data are available for the majority of early Cambrian regions of the Siberian Craton, so we use the distribution of metazoan benthos as a proxy for the presence of shallow, habitable (oxic), sea floor. We also consider the distribution of the heavily calcified hyoliths, molluscs, and calcareous SSFs. These data are integrated with geological facies maps, chemostratigraphic information (positive δ^13^C_carb_ excursions 5p, 6p, and II to VII), biozones, and submeter-scale lithostratigraphic subdivisions recalculated to the same stratigraphic scale, over 23 Ma from the Fortunian to Cambrian stage 4 (lowermost Tommotian 1 to Toyonian 3/Amgan on the Siberian stratigraphic scale), from 528 to 505 Ma ago (Fig. 2A).

## Supplementary Materials

### This PDF file includes:

Stratigraphic Notes Figs. S1 and S2 Table S1 Legend for supplementary data Legend for fig. S1, full version References

### Other Supplementary Material for this manuscript includes the following:

Supplementary Data

Fig. S1, full version
