# Supplementary material for: Cambrian radiation speciation events driven by sea level and redoxcline changes on the Siberian Craton
Source: Sci Adv. 2023 Jun 16;9(24):eadh2558. doi: 10.1126/sciadv.adh2558 (PMC10275598; doi:10.1126/sciadv.adh2558)
Supplement: Supplementary file 1 — Stratigraphic Notes Figs. S1 and S2 Table S1 Legend for supplementary data Legend for fig. S1, full version References [file sciadv.adh2558_sm.pdf]

Science Advances
AAAS

Supplementary Materials for

Cambrian radiation speciation events driven by sea level and redoxcline changes on the Siberian Craton

Andrey Yu. Zhuravlev *et al.*

Corresponding author: Rachel A. Wood, rachel.wood@ed.ac.uk

Sci. Adv. **9**, eadh2558 (2023)
DOI: 10.1126/sciadv.adh2558

The PDF file includes:

Stratigraphic Notes
Figs. S1 and S2
Table S1
Legend for supplementary data
Legend for fig. S1, full version
References

Other Supplementary Material for this manuscript includes the following:

Supplementary Data
Fig. S1, full version

Supplementary Information

Cambrian radiation speciation events driven by sea level and redoxcline changes on the Siberian Craton

Andrey Yu. Zhuravlev, Rachel A. Wood, Fred T. Bowyer

Stratigraphic Notes
Figure S1
Figure S2
Table S1
Supplementary Data
Auxiliary figure file, Figure S1.

Stratigraphic Notes

Rozanov et al. *(43)* indicated an archaeocyath occurrence in the uppermost Sukharikha Formation on the Sukharikha River (Igarka bank). Selinde and Sukharikha rivers were on opposite sides of the Siberian craton (Figs. 1a, S1), suggesting a simultaneous appearance of the same several species in two remote areas. However, none of these Sukharikha fossils were ever photographed, nor have any previous or subsequent studies confirmed archaeocyaths in the Sukharikha Formation *(56,59,83–85)*. Re-study of thin sections from the Rozanov et al. *(43)* collection have confirmed the absence of archaeocyaths in this collection from the uppermost Sukharikha Formation.

Fig. S1. Outcrops and boreholes stratigraphy and distribution. High-resolution lithostratigraphic sections and maps showing regions with distribution of archaeocyath outcrops and boreholes. See also separate file for high-resolution image.

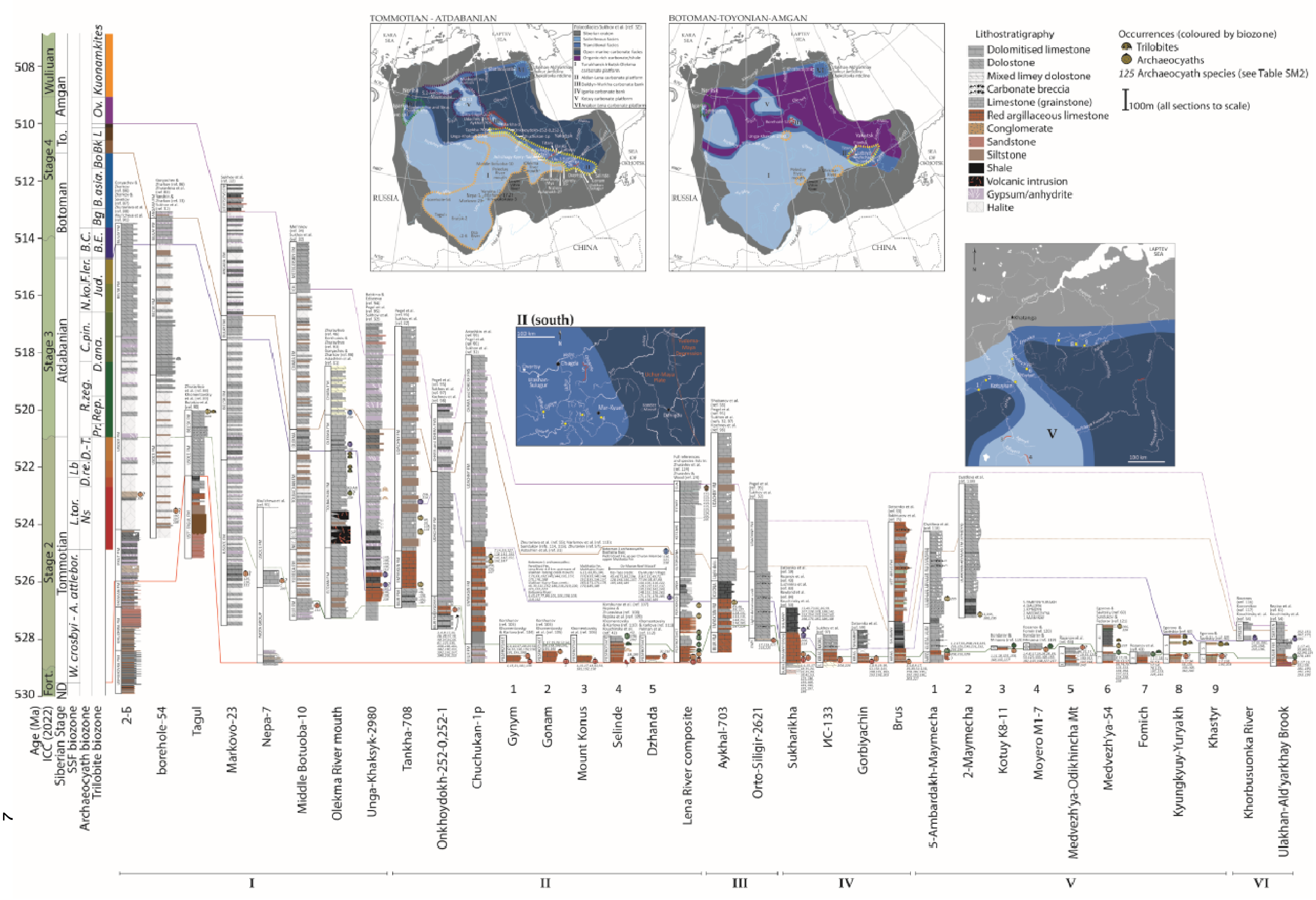

TOMMOTIAN - ATDABANIAN
BOTOMAN-TOYONIAN-AMGAN
II (south)
V
Lithostratigraphy
Dolomitised limestone
Dolostone
Mixed limey dolostone
Carbonate breccia
Limestone (grainstone)
Red argillaceous limestone
Conglomerate
Sandstone
Siltstone
Shale
Volcanic intrusion
Gypsum/anhydrite
Halite
Occurrences (coloured by biozone)
Trilobites
Archaeocyaths
125 Archaeocyath species (see Table SM2)
100m (all sections to scale)
Age (Ma)
ICC (2022)
Siberian Stage
SSF biozone
Archaeocyath biozone
Trilobite biozone
Fort.
Stage 2
Stage 3
Stage 4
Wuliuan
ND
Tommotian
Atdabanian
Botoman
To.
Amgan
W. crosbyi - A. attlebor.
L.tor.
Ns
Lb
D.re.
D.-T.
Pr.
Rep.
R.zeg.
D.ana.
C.pin.
N.ko.
F.ler.
Jud.
B.E.
B.C.
Bg
B.asia.
Bo
Bk
L
Ov.
Kuonamkites
508
510
512
514
516
518
520
522
524
526
528
530
2-5
borehole-54
Tagul
Markovo-23
Nepa-7
Middle Botuoba-10
Olekma River mouth
Unga-Khaksyk-2980
Tankha-708
Onkhoydokh-252-0,252-1
Chuchukan-1p
1 Gynym
2 Gonam
3 Mount Konus
4 Selinde
5 Dzhanda
Lena River composite
Aykhal-703
Orto-Siligir-2621
Sukharikha
ИС-133
Gorbiyachin
Brus
1 5-Ambardakh-Maymecha
2 2-Maymecha
3 Kotuy K8-11
4 Moyero M1-7
5 Medvezh'ya-Odikhincha Mt
6 Medvezh'ya-54
7 Fomich
8 Kyungkyuy-Yuryakh
9 Khastyr
Khorbusuonka River
Ulakhan-Ald'yarkhay Brook
I
II
III
IV
V
VI

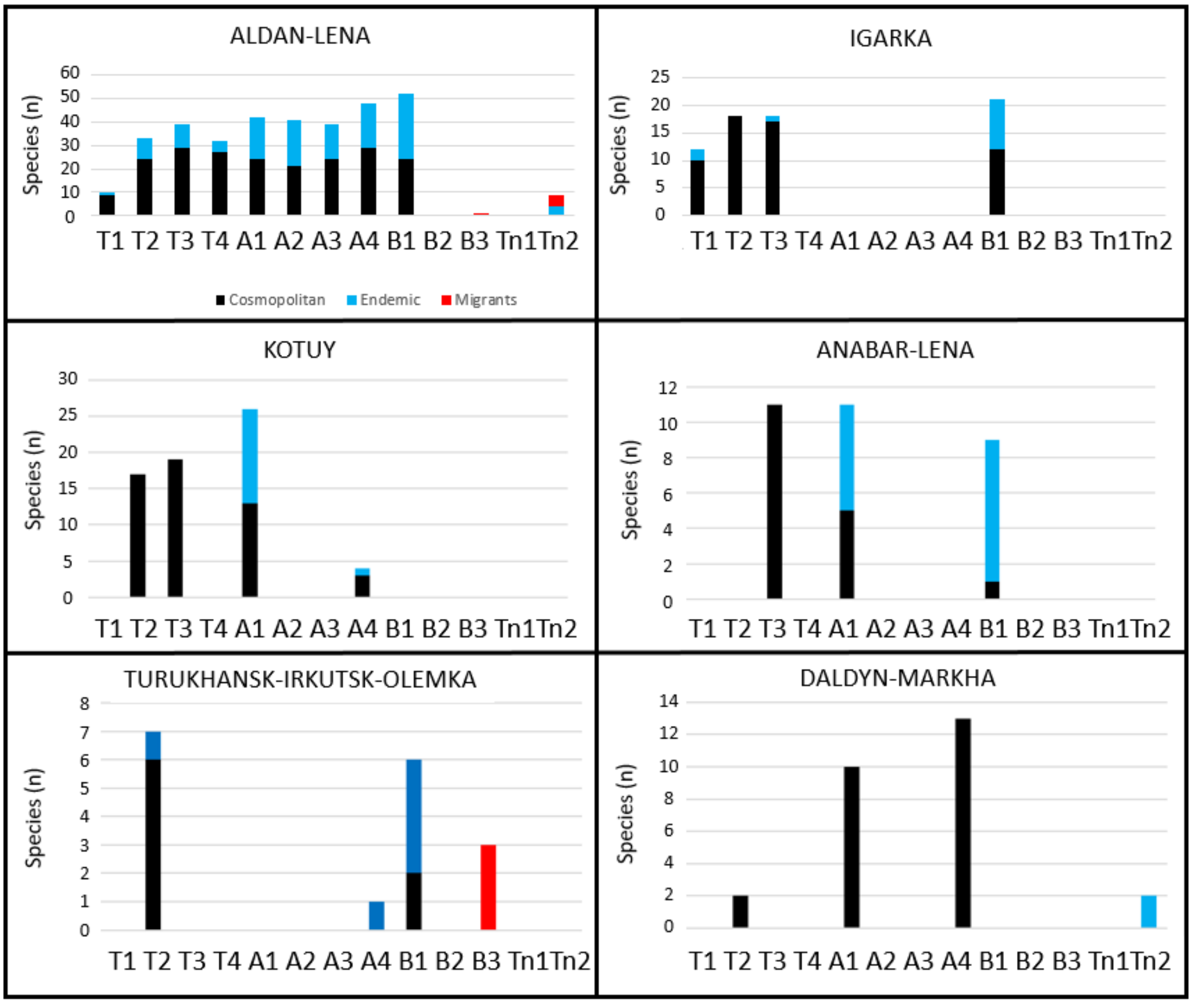

Fig. S2. Archaeocyath species distribution on Siberian Craton. Distribution of cosmopolitan, endemic, and migrant archaeocyath species in each region on the Siberian Craton through the early Cambrian.

Table S1: List of regions and localities (outcrop and borehole) with archaeocyath occurances on the Siberian Craton. Endemic species indicated in region's colour. T = Tommotian; A = Atdabanian; B = Botoman; Tn = Toyonian

1. Turukhansk-Irkutsk-Olekma carbonate platform

Cis-Sayan area

2-Б borehole, Tagna village on the Oka River, Angara River left tributary *(86–88)*:

T2 – Usol'e Formation, depth 1453.0–1420.0 m: *Nochoroicyathus* sp.

Borehole 54, Troitsk anticline *(32,33,86,88)*:

T2 – Usol'e Formation, depth 1454.8–1454.2 m:

Cambrocyathellus sp., *Sakhacyathus subartus, Dictyocyathus* sp.

Tagul River *(88–90)*:

A4 – Bel'sk Formation, 115 m above the base:

Nochoroicyathus cf. *osensis*.

Angara-Nepa area

Markovo 23-p borehole *(32)*:

T2 – Usol'e Formation, depth 2552–2481 m:

Nochoroicyathus ex gr. virgatus, *Sibirecyathus* cf. *suvorovae*, *Tumulocyathus kotuyikensis*.

Nepa 7 borehole on the Kulinda River, Nizhnyaya Tunguska River left tributary *(91)*:

T2 – Usol'e Formation, depth 2298.5–2238.4 m:

Nochoroicyathus cf. *osensis*.

Bratsk 2 borehole *(91)*:

T2 – Usol'e Formation, depth 2991.8 m:

Nochoroicyathus sp.

Markovo 17 borehole *(88)*:

T2 – Usol'e Formation, depth 2432.6 m:

Nochoroicyathus ex gr. *virgatus*.

Markovo 27 borehole *(88)*:

T2 – Usol'e Formation, depth 2177.5 m:

Sibirecyathus cf. *suvorovae*.

Krivolutskaya 3 borehole *(88)*:

T2 – Bel'sk Formation, depth 1760 m:

Nochoroicyathus ex gr. tkatschenkoi.

Osa River borehole *(92)*:

T2 – Usol'e Formation, depth 701.5–695.7 m:

Nochoroicyathus osensis.

Yarakta 12 borehole *(91)*:

T2 – Usol'e Formation, depth 2259 m:

Nochoroicyathus sp.

Botuoba area

Middle Botuoba-10 borehole *(32,34)*:

T2 – Bilir Formation, depth 1512–1488 m:

Rotundocyathus cf. novus.

Lower Vitim River *(46,88)*:

A3-A4 ? – Tolbachan Formation, m:

Rotundocyathus peleduicus.

B3 – Olekma Formation, upper part:

Claruscoscinus sp*, Archaecyathus kusmini?*.

Nokhtuysk section, Olekma River mouth *(31,46,86,93)*:

A3-A4 ? – Tolbachan Formation, middle part:

Nochoroicyathus cf. osensis.

B1 – Olekma Formation, lower part:

Irinaecyathus jadwigae.

B3 – Chara Formation, lower part:

Cellicyathus sp.

Syudzha area

Unga-Khaksyk 2980 borehole *(32,94,95)*:

B1 ? – Sygdakh Formation, depth 3044.9–3030.4 m:

Nochoroicyathus cf. *osensis, Fallocyathus apheles, F. accomodatus, Dictyocyathus bobrovi*.

B1 – Sygdakh Formation, depth 2997.1–2986.0 m:

Rhabdolynthus conicus, Dokidocyathus sp., *Fallocyathus apheles, F. accomodatus, Erismacoscinus aculeatus*.

2. Daldyn-Markha carbonate bank

Orto-Siligir 2621 borehole *(32,95)*:

T2 ? – Emyaksin Formation, depth 1280–1277 m:

Archaeolynthus sp., *Nochoroicyathus tkatschenkoi, Tumulocyathus* sp., *Erismacoscinus* sp.; radiocyath *Gonamispongia ignorabilis*.

Markha 1 borehole *(96)*:

T2 ? – Emyaksin Formation, depth 1440.8–1436.8 m:

Nochoroicyathus mirabilis, N. sunnaginicus, Dictyocyathus sp.

A1 – Emyaksin Formation, depth 1413.3–1403.6 m:

Archaeolynthus sp., *Nochoroicyathus mirabilis, N. sublenaicus, N. 'arteintervallum', Rotundocyathus? grandis, Leptosocyathus polyseptus, Baikalocyathus? sp., Tumulocyathus kotuyikensis, Erismacoscinus oymuranensis*, *Korshunovicyathus melnikovi, Sakhacyathus subartus, Dictyocyathus bobrovi, Dictyosycon gravis*.

Aykhal 703 borehole *(32,58,95,97,98)*:

A4 – Emyaksin Formation, depth 1738–1701 m:

Nochoroicyathus kokoulini, N. biohermicus, Rotundocyathus sublenaicus, Tumulocyathus tuberculatus, Plicocyathus platiseptatus, Geocyathus botomaensis, G. latini, Japhanicyathus

genurosus, Carinacyathus pinus, Fansycyathus lermontovae, Tumulocoscinus atdabanensis, Fransuasaecyathus elegans, Coscinocyathus isointervallumus; radiocyath *Girphanovella* sp.

Udachny 2531 borehole *(32)*:

Emyaksin Formation, depth 1700-1691 m:

A4 – *Nochoroicyathus kokoulini, Rotundocyathus sublenaicus, Plicocyathus platiseptatus, Geocyathus botomaensis, G. latini, Coscinocyathus isointervallumus*.

Borehole 122 *(32)*:

Tn2 – Udachny Formation, depth 1197.5 m:

Tegerocyathus edelsteini?, Archaeocyathus okulitchi

3. Aldan-Lena area of the Anabar-Sinsk reef margin

Aldan-Lena area (except for Tommotian-Atdabanian occurrences of the middle Aldan and Lena rivers listed in 21)

Chuchukan-1p borehole *(32,95,99)*:

A4 ? – Emyaksin Formation, depth 3277–3268 m:

Tumuliolynthus sp., *Nochoroicyathus* sp., *Tumulocyathus* sp.

A4 – Emyaksin Formation, depth 3266–3254 m:

Propriolynthus vologdini, Batschykicyathus angulosus, Rotundocyathus sublenaicus, Tumulocyathus tuberculatus, Plicocyathus platiseptatus, Geocyathus botomaensis, G. latini, Jakutocarinus jakutensis, Gagarinicyathus ethmophylloides, Carinacyathus pinus, Erismacoscinus batchykensis, Coscinocyathus isointervallumus.

Tankha 708 borehole *(95,97)*:

A4 – Emyaksin Formation, depth 2614–2604 m:

Nochoroicyathus kokoulini, N. heteroporatus, Plicocyathus platiseptatus, Geocyathus latini, Coscinocyathus isointervallumus.

A4 – Udachny Formation, depths 2379–2369 m; 2309–2300 m:

Nochoroicyathus kokoulini, Rotundocyathus sublenaicus, Plicocyathus platiseptatus, Geocyathus botomaensis.

B1 ? – Udachny Formation, depth 2249–2240 m:

Nochoroicyathus sp., *Geocyathus* sp.

Onkhoydokh-252-0 borehole *(95,97,98)*:

T2 ? – Sygdakh Formation, depth 1870.4–1830.0 m:

Archaeolynthus polaris, Tumuliolynthus primigenius, Dokidocyathus subrarus, Nochoroicyathus mirabilis, N. occultatus, N. tkatschenkoi, N. turbidus, N. multiformis, N. ridiculus, N. microtumulus, Rotundocyathus grandis, R. spinosus, R. indistinctus, Sibirecyathus onkhoydokh, Tumulocyathus kotuyikensis, Sclerocyathus australis, Erismacoscinus rojkovi, Retecoscinus sakhaensis, Capsulocyathus petri, Cryptoporocyathus junicanensis, Cambrocyathellus robustus, C. tschuranicus, C. proximus, Okulitchicyathus discoformis, Sakhacyathus subartus, Dictyocyathus translucidus, Spinosocyathus maslennikovae; radiocyath *Gonamispongia ignorabilis*.

Nizhniy Kuranakh-27 borehole *(100)*
T2 – Pestrotsvet Formation, 50 m above the base of the formation:

not described.

Gorely-16 borehole *(100)*
T1-T2 – Pestrotsvet Formation, 0-50 m above the base of the formation:

not described.

Kurum-Kyunkyu-26 borehole *(100)*
T1-T2 – Pestrotsvet Formation, 0-50 m above the base of the formation:

not described.

Ulu-3 borehole *(100)*
T1-T2 – Pestrotsvet Formation, 0-50 m above the base of the formation:

not described.

Amga River *(31,101,102)*:

B3 – Olekma Formation, 20-30 m above the base of the formation:

Cellicyathus sp., *Archaecyathus kusmini?*.

Tn2 – Khomustakh Formation, middle and upper parts:

Irinaecyathus lenaicus, Kiwicyathus? egorovae, Erbocyathus heterovallum, Tegerocyathus edelsteni?, Archaecyathus kusmini?.

Gynym River *(103,104)*:

T1 – Pestrotsvet Formation, Sunnagin Member, 0-3.8 m above the base:

Archaeolynthus polaris, Nochoroicyathus sunnaginicus, N. vulgaris, Cryptoporocyathus junicanensis, *Okulitchicyathus discoformis*.

T2 – Pestrotsvet Formation, 23.8-36.3 m above the base of the formation:

Archaeolynthus polaris, Dokidocyathus regularis, D. konsimilis, Nochoroicyathus tkatschenkoi, N. simplex, N. mirabilis, Rotundocyathus? anabarensis, *R. spinosus, R. anomalus, Erismacoscinus rojkovi, Cambrocyathellus robustus, C. tschuranicus, C. proximus, Okulitchicyathus discoformis, Dictyocyathus translucidus*.

Gonam River ***(103,105)*****:**

T1 – Pestrotsvet Formation, Sunnagin Member, 0-3 m above the base:

Archaeolynthus polaris.

T3 – Pestrotsvet Formation, 51-57.5 m above the base of the formation:

Archaeolynthus polaris, Nochoroicyathus tkatschenkoi, N. mirabilis, N. turbidus, Rotundocyathus? anabarensis, *R. anomalus, R. spinosoporosus, Erismacoscinus rojkovi, Cambrocyathellus tschuranicus, C. proximus, Okulitchicyathus discoformis, Dictyocyathus translucidus*.

Mount Konus, section 8 ***(106)*****:**

T1 – Pestrotsvet Formation, Sunnagin Member, 2-3 m above the base:

Archaeolynthus polaris, Nochoroicyathus sunnaginicus, N. virgatus, N. tkatschenkoi, N. similis, Rotundocyathus? anabarensis, *Cryptoporocyathus junicanensis*, *Cambrocyathellus robustus, Dictyocyathus translucidus*.

Selinde River ***(42,107–110)*****:**

T1 – Pestrotsvet Formation, Sunnagin Member, 2.7 m above the base:

undetermined archaeocyath fragments.

T2 – Pestrotsvet Formation, 5 m above the base of the formation:

Nochoroicyathus mirabilis, Cambrocyathellus tschuranicus.

A1 – Pestrotsvet Formation, 122-123 m above the base of the formation:

Nochoroicyathus 'arteintervallum', Neoloculicyathus sibiricus, Dictyosycon gravis.

Dzhanda River ***(111,112)*****:**

T2 – Pestrotsvet Formation, 546-48 m above the base of the formation:

Archaeolynthus sp., *Nochoroicyathus similis*.

Middle Lena River, Mukhatta River ***(31,52)*****:**

B1 – Mukhatta Formation, in the lower 30-40 m of the formation:

Tumuliolynthus karakolensis, Rotundocyathus lenaicus, Sibirecyathus polysynapticulosus, Taylorcyathus subtaylori, Degeletticyathus galushkoi, Robertocyathus meshkovae, Ladaecyathus sublimbatus, Rossocyathella shenfili, Fansycyathus lermontovae, Carinacyathus squamosus, Squamosocyathus taumatus, Botomocyathus zelenovi, B. astrumus, Erismacoscinus fimbriatulus, Antoniocoscinus vsevolodi, Agyrekocyathus gratus, Rozanovicyathus alexi, Muchattocyathus sibiricus, Fransuasaecyathus elegans, Coscinocyathus latus.

Middle Lena River, Oy-Muran Village ***(52,113)*****:**

B1 – Oy-Muran Reef Massif:

Archaeolynthus 'nalivkini', Tumuliolynthus karakolensis, Propriolynthus vologdini, Rhabdolynthus conicus, Dokidocyathella incognita, Nochoroicyathus biohermicus, Rotundocyathus novus, R. jakshini, Robustocyathellus erbocyathoides, Taylorcyathus subtaylori, Gordonicyathus apprimus, Trininaecyathus macroporus, Degeletticyathus galushkoi, Zonacyathus? einastoi, Robertocyathus meshkovae, Ladaecyathus sublimbatus, Isiticyathus ultrus, Plicocyathus platiseptatus, Geocyathus latini, G. krasnopeevae, Japhanicyathus genurosus, Rossocyathella ninaekosti, Fallocyathus dubius, Fansycyathus lermontovae, Jakutocarinus jakutensis, Squamosocyathus taumatus, Botomocyathus zelenovi, Erismacoscinus fimbriatulus, Antoniocoscinus vsevolodi, Agyrekocyathus gratus, Muchattocyathus sibiricus, Fransuasaecyathus elegans, *Coscinocyathus marocanoides, C. latus*.

Middle Lena River, Kisi-Taas Creek ***(52,54)*****:**

B1 – Oy-Muran Reef Massif:

Nochoroicyathus biohermicus, N. consuetus, Rotundocyathus novus, Sibirecyathus polysynapticulosus, Zonacyathus? einastoi, Plicocyathus platiseptatus, Fansycyathus lermontovae, Botomocyathus zelenovi, B. astrumus, Fransuasaecyathus elegans, *Coscinocyathus marocanoides, C. latus*.

Middle Lena River, 4.8 km above the Ulakhan-Taryng Creek mouth ***(54)*****:**

B1 – Perekhod Formation, 40 m above the base of the formation:

Propriolynthus vologdini, Rotundocyathus novus, Sibirecyathus polysynapticulosus, Geocyathus krasnopeevae, Rossocyathella shenfili, Fallocyathus dubius, Squamosocyathus taumatus, Botomocyathus astrumus, Agyrekocyathus grigorievi, Tumulocoscinus atdabanensis, Coscinocyathus marocanoides.

Middle Lena River, Ulakhan-Kyyry-Taas Creek ***(113)*****:**

B1 – Perekhod Formation, member III, 6 m above the base of the member:

Nochoroicyathus biohermicus, Rotundocyathus ex gr. *lenaicus, Geocyathus latini, Jakutocarinus?* sp., *Tumulocoscinus* sp.

B1 – Perekhod Formation, member IV, 14 m above the base of the member:

Archaeolynthus sp.*, Rotundocyathus lenaicus, Trininaecyathus* sp.*, Plicocyathus* sp.*, Carinacyathus squamosus, Coscinocyathus marocanoides*.

Buotama River ***(52,113,114)*****:**

B1 – Perekhod Formation, member IV, 3 m above the base of the member:

Tumuliolynthus karakolensis, Nochoroicyathus biohermicus, N. facetus, Rotundocyathus jakshini, Trininaecyathus macroporus, Baikalocyathus rossicus, Degeletticyathus galushkoi, Zonacyathus? einastoi, Irinaecyathus 'ratus', Robertocyathus alexseevi, Carinacyathus squamosus.

Buotama River ***(52,113,114)*****:**

B1 – Perekhod Formation, member IV, 3 m above the base of the member:

Tumuliolynthus karakolensis, Nochoroicyathus biohermicus, N. facetus, Rotundocyathus jakshini, Trininaecyathus macroporus, Baikalocyathus rossicus, Degeletticyathus galushkoi, Zonacyathus? einastoi, Irinaecyathus 'ratus', Robertocyathus alexseevi, Carinacyathus squamosus.

Buotama River, 100 m above the Kyra-Taas River mouth, section 430 ***(115)*****:**

B3 – Pestrotsvet Formation, Churan Member, upper part:

Robertocyathus meshkovae, Botomocyathus zelenovi.

Buotama River, 100 m above the Kyra-Taas River mouth, section 444 ***(115)*****:**

B3 – Mukhatta Formation, upper part:

Erbocyathus heterovallum.

Middle Lena River, Elanka Village ***(113)*****:**

Tn2 – Elanka Formation, 8 m above the base of the formation:

Irinaecyathus 'grandiperforatus', I. shabanovi, I. lenaicus, Erbocyathus heterovallum, Tegerocyathus edelsteini?, T. ketemensis, Kiwicyathus? egorovae, Archaeocyathus kusmini, A. okulitchi.

Tn2 – Elanka Formation, 11.5 above the base of the formation:

Kiwicyathus? egorovae.

4. Anabar-Lena carbonate platform

Ulakhan-Ald'yarkhay Brook, Bulkur anticline *(57,61)*:

T3 – Tyuser Formation, 23.5-25.5 m above the base of the formation:

Archaeolynthus polaris, Nochoroicyathus tkatschenkoi, N. mirabilis, Rotundocyathus? anabarensis, *Erismacoscinus fedorovi, Cryptoporocyathus junicanensis*, *Cambrocyathellus robustus, C. tschuranicus, C. proximus, Okulitchicyathus discoformis, Dictyocyathus translucidus*.

A1 – Tyuser Formation, 39.5-44.5 m above the base of the formation:

Archaeolynthus polaris1, Nochoroicyathus tkatschenkoi, N. subturbidus, Rotundocyathus? grandis, *R. orbus, R. tetracyclicus, R. apertus, Taylorcyathus eximius, Lenocyathus lenaicus, Retecoscinus proximus, Korshunovicyathus melnikovi*.

B1 – Sekten Formation, 0.2 m above the base of the formation:

Carinacyathus squamosus, C. minaevae, Erismacoscinus vinogradovi, Dentatocoscinus sektensis, Loculicyathus legitimus.

Tyuser Brook, Bulkur anticline *(61)*:

A1 – Tyuser Formation, middle part:

Nochoroicyathus cf. gigantoporus, Rotundocyathus? grandis, *Lenocyathus lenaicus, Korshunovicyathus melnikovi*.

Chekurovka Village, Chekurovka anticline *(31,61)*:

T3 – Tyuser Formation, 10-20 m above the base of the formation:

Archaeolynthus polaris, Nochoroicyathus tkatschenkoi, N. mirabilis, Rotundocyathus? anabarensis, *Erismacoscinus fedorovi, Cryptoporocyathus junicanensis*, *Cambrocyathellus robustus, C. tschuranicus, C. proximus, Okulitchicyathus discoformis, Dictyocyathus translucidus*.

A1 – Tyuser Formation, 21-30 m above the base of the formation:

Archaeolynthus polaris1, Rotundocyathus? anabarensis, *R.? grandis*, *R. tetracyclicus, R. apertus, Retecoscinus proximus*.

B1 – Sekten Formation, 0-5 m above the base of the formation:

Carinacyathus squamosus, C. minaevae, Erismacoscinus vinogradovi, Dentatocoscinus sektensis, Loculicyathus legitimus.

Saakhtany River, Chekurovka anticline *(61)*:

T3 – Tyuser Formation, lower part:

Archaeolynthus sp., *Nochoroicyathus tkatschenkoi, N. mirabilis, Rotundocyathus* sp., *Cryptoporocyathus junicanensis*, *Okulitchicyathus discoformis, Dictyocyathus translucidus*.

Khorbusuonka River, Olenek Uplift *(57,116,117)*:

B1 – Erkeket Formation, 50 m below the base of the Kuonamka Formation:

Nochoroicyathus ex gr. *sukharichensis*, *Carinacyathus squamosus, Robertocyathus polaris, Mattajacyathus arduus, Gloriosocyathus permultus*.

5. Igarka carbonate bank
ИС-133 borehole *(97)*:

T2 – Krasny Porog Formation, depth 154.8–150.2 m:

Nochoroicyathus sp., *Rotundocyathus?* sp.

Gorbiyachin River *(59)*:

T1 – Krasny Porog Formation, lower 3.5 m:

Archaeolynthus polaris1, Dokidocyathus regularis, D. subrarus, Nochoroicyathus mirabilis, N. occultatus, Rotundocyathus? anabarensis, Cambrocyathellus tschuranicus, Okulitchicyathus discoformis, Dictyocyathus translucidus.

T2 ? – Krasny Porog Formation, 3.5-5 m above the base:

Archaeolynthus polaris1, Nochoroicyathus mirabilis, N. occultatus, Rotundocyathus? anabarensis, Erismacoscinus rojkovi, Cryptoporocyathus junicanensis, Cambrocyathellus robustus, C. tschuranicus, C. proximus, Okulitchicyathus discoformis, Dictyocyathus translucidus, Spinosocyathus maslennikovae.

Brus River *(59,75)*:

T2 ? – Krasny Porog Formation, lower 2.5 m:

Archaeolynthus polaris1, Tumuliolynthus primigenius, Dokidocyathus regularis, D. subrarus, Nochoroicyathus mirabilis, N. occultatus, N. tkatschenkoi, Rotundocyathus? anabarensis, Tumulocyathus kotuyikensis, Erismacoscinus rojkovi, Cryptoporocyathus junicanensis, Cambrocyathellus robustus, C. tschuranicus, Okulitchicyathus discoformis, Dictyocyathus translucidus, Spinosocyathus maslennikovae; coralomorph *Cysticyathus tunicatus*.

Sukharikha River *(43,56,59,83,84)*:

T1 – Krasny Porog Formation, 0-1 m above the base of the formation:

Archaeolynthus polaris1, Nochoroicyathus sunnaginicus, N. virgatus, N. igarkaensis, N. dragunovi, Cryptoporocyathus junicanensis.

T2 – Krasny Porog Formation, 1-5 m above the base of the formation:

Archaeolynthus polaris1, Nochoroicyathus sunnaginicus, Cambrocyathellus tschuranicus, C. proximus, Dictyocyathus translucidus.

T3 – Krasny Porog Formation, 10.5-19 m above the base of the formation:

Archaeolynthus polaris1, Tumuliolynthus primigenius, Nochoroicyathus tkatschenkoi, Nochoroicyathus mirabilis, N. aldanicus, N. occultatus, N. 'arteintervallum', N. pseudoccultatus, Rotundocyathus? anabarensis, Tumulocyathus kotuyikensis, Sclerocyathus australis, Erismacoscinus rojkovi, Retecoscinus sakhaensis, Cryptoporocyathus junicanensis, Cambrocyathellus tschuranicus, C. proximus, Sakhacyathus subartus, Dictyocyathus translucidus.

B1 – Krasny Porog Formation, upper 30 m:

Nochoroicyathus sp., *Plicocyathus* sp.

B1 – Shumny Formation, lower part:

Rhabdolynthus conicus, Dokidocyathella incognita, Nochoroicyathus sucharichensis, Rotundocyathus plumospinosus, Stapicyathus stapiporus, Sibirecyathus sokolovi, Trininaecyathus macroporus, Irinaecyathus inoratus, Plicocyathus plastiseptatus, Geocyathus latini, Tumulifungia datzenkoi, Gagarinicyathus ethmophylloides, Carinacyathus squamosus, Hupecyathellus chouberti, Squamosocyathus taumatus, Botomocyathus zelenovi, Erismacoscinus fimbriatulus, E. savitzkii, Agyrekocyathus grigorievi, Schumnyicyathus validus, Coscinocyathus marocanoides.

6. Kotuy carbonate platform

Ambradakh and Maymecha rivers' confluence borehole *(118)*:

T2 ? – Medvezh'ya Formation, depth 503.8-491.3 m:

undetermined archaeocyath fragments.

T2 – Medvezh'ya Formation, depth 491.8-476.0 m:

Cryptoporocyathus sp.

T2 – Medvezh'ya Formation, depth 476.0-466.3 m:

Dokidocyathus sp., *Dictyocyathus* sp.

A1 ? – Kugda-Yuryakh Formation, depth 466.0-464.3 m:

Archaeolynthus cf. *polaris, Dokidocyathus* sp.

A1 ? – Kugda-Yuryakh Formation, depth 450.0-449.3 m:

Dokidocyathus sp., *Nochoroicyathus* cf. *sunnaginicus, N. tkatschenkoi, N.* ex gr. *grandis, Rotundocyathus* sp., *Tumulocyathus?* sp., *Korshunovicyathus?* sp.

A1 ? – Kugda-Yuryakh Formation, depth 447.0-445.8 m:

Archaeolynthus polaris2, Rotundocyathus anabarensis, Tumulocyathus cf. *kotuyikensis*, *Erismacoscinus* sp., *Korshunovicyathus?* sp.

A1 ? – Kugda-Yuryakh Formation, depth 443.8-439.3 m:

Archaeolynthus polaris, Erismacoscinus sp., *Korshunovicyathus?* sp., *Dictyocyathus* sp.

A4 – Ulakhan-Arymas Formation, depth 209.1-205.0 m:

Taylorcyathus? sp.

/trilobite *Pagetiellus lenaicus* (**A3-B1**) is present in this formation only, depth 325.8-318.0 m/

Maymecha River borehole *(118)*:

A4 – Ulakhan-Arymas Formation, depth 493.6-487.3 m:

Archaeolynthus '*nalivkini*', *Nochoroicyathus* sp., *Squamosocyathus taumatus, Coscinocyathus* ex gr. *isointervallumus.*

Kotuy River, sections K8-11 *(119)*:

T3 – Medvezh'ya Formation, 0-6 m above the base of the section:

Archaeolynthus polaris1, Dokidocyathus lenaicus, *Nochoroicyathus turbidus, Tumulocyathus kotuyikensis, Erismacoscinus rojkovi, Retecoscinus sakhaensis, Korshunovicyathus melnikovi;* coralomorph *Cysticyathus tunicatus*.

Moyero River, sections M1-7 *(119,120)*:

T3 – Medvezh'ya Formation, 1-5.6 m above the base of the section:

Archaeolynthus polaris1, Dokidocyathus regularis, Tumuliolynthus primigenius, Nochoroicyathus tkatschenkoi, N. mirabilis, N. turbidus, N. ridiculus, N. occultatus, Tumulocyathus kotuyikensis, Erismacoscinus rojkovi, Retecoscinus sakhaensis, Cambrocyathellus robustus, Sakhacyathus subartus, Dictyocyathus translucidus; coralomorph *Cysticyathus tunicatus*.

T3 – Medvezh'ya Formation, 5.6-16.5 m above the base of the section:

Nochoroicyathus turbidus, Rotundocyathus? anabarensis, Tennericyathus sp., *Erismacoscinus rojkovi, Korshunovicyathus melnikovi, Sakhacyathus subartus, Dictyocyathus translucidus*.

Medvezh'ya River, Odikhincha Mount *(43)*:

T2 ? – Medvezh'ya Formation, 40 m above the base of the formation:

Archaeolynthus polaris1, Nochoroicyathus vulgaris, N. tkatschenkoi, N. mirabilis, Rotundocyathus? anabarensis, R. ex gr. *grandis, Tumulocyathus kotuyikensis, Cambrocyathellus robustus, C. tschuranicus, Sakhacyathus subartus, Dictyocyathus translucidus*.

Medvezh'ya River, above the Daldyn River mouth section, section 54 *(60,121)*:

A1 – Medvezh'ya Formation, 40-50 m above the base of the formation:

Archaeolynthus polaris2, Dokidocyathella cf. *incognita, Nochoroicyathus tkatschenkoi, N. mirabilis, N. heteroporatus, Rotundocyathus? anabarensis, R. sublenaicus, R. alexandrae, R.*

dotatus, R. shabanovi, Baikalocyathus sp., *Tumulocyathus kotuyikensis, Plicocyathus* sp., *Geocyathus botomanensis, Erismacoscinus* sp., *Korshunovicyathus melnikovi, Sakhacyathus subartus, Neoloculicyathus sibiricus*.

A4 – Daldyn Formation, at the base:

Erismacoscinus? sp.

A4 – Daldyn Formation, 90 m above the base:

Nochoroicyathus sp., *Rotundocyathus* sp.

Kotuy River, D'yama-Yuryakh and Ebe-Yur'yakh creeks *(122)*:

A1 – Kyndyn Formation, lower part:

Archaeolynthus polaris2, Nochoroicyathus kotuyikensis, Rotundocyathus? grandis, R. minaevae, R. kashinae, Tennericyathus odichinchensis, Taylorcyathus milashevae, Tumulocyathus kotuyikensis, T. danieli, Korshunovicyathus melnikovi, Fransuasaecyathus subtumulatus, Sakhacyathus subartus, Neoloculicyathus sibiricus.

Kotuy River, Chomp-Yuryakh Creek *(123)*:

A1 – Kyndyn Formation, 25-30 m above the base of the formation:

Kotuyicoscinus minaevae.

Fomich River *(43)*:

A1 – Medvezh'ya Formation, 60-70 m above the base of the formation:

Archaeolynthus polaris, Nochoroicyathus tkatschenkoi, N. turbidus, N. kotuyikensis, Rotundocyathus? anabarensis, R. dotatus, R. moori, Ajacicyathus cautus, Tumulocyathus kotuyikensis, Plicocyathus sp., *Erismacoscinus* sp., *Fransuasaecyathus* sp., *Sakhacyathus subartus*.

Kyungkyuy-Yuryakh River, sections 128-132 *(60)*:

T2 ? – Medvezh'ya Formation, 30 m above the base of the formation:

Archaeolynthus polaris, Nochoroicyathus tkatschenkoi, N. simplex, Rotundocyathus? anabarensis, Tumulocyathus kotuyikensis, Erismacoscinus rojkovi, Retecoscinus sakhaensis, Cambrocyathellus robustus, Spinosocyathus maslennikovae.

Khastyr River, sections 1208, 1210 *(60)*:

T2 ? – Medvezh'ya Formation, 30 m above the base of the formation:

Nochoroicyathus tkatschenkoi, Erismacoscinus rojkovi, Cambrocyathellus robustus, Spinosocyathus maslennikovae.

Supplementary Data: Data set of temporal and spatial distributions of archaeocyath species on the Siberian Craton. Total species data, and cosmopolitan and endemic archaeocyath species by region; Determined ancestor-descendent pairs, and speciation modes, of archaeocyath species on the Siberian Craton; Full updated $\delta^{13}C_{carb}$ age model; references.

Auxiliary figure file, Figure S1. Fig. S1. Outcrops and boreholes stratigraphy and distribution. High-resolution lithostratigraphic sections and maps showing regions with distribution of archaeocyath outcrops and boreholes. See also separate file for high-resolution image.

REFERENCES AND NOTES

1. D. H. Erwin, J. W. Valentine, *The Cambrian Explosion: The Construction of Animal Biodiversity* (Greenwood Village, 2013).

2. E. E. Saupe, C. E. Myers, A. Townsend Peterson, J. Soberón, J. Singarayer, P. Valdes, H. Qiao, Spatio-temporal climate change contributes to latitudinal diversity gradients. *Nat. Ecol. Evol.* **3**, 1419–1429 (2019).

3. S. R. Palumbi, Genetic divergence, reproductive isolation, and marine speciation. *Annu. Rev. Ecol. Syst.* **25**, 547–572 (1994).

4. J. J. Álvaro, P. Ahlberg, L. E. Babcock, O. L. Bordonaro, D. K. Choi, R. A. Cooper, G. Kh. Ergaliev, I. W. Gapp, M. Ghobadi Pour, N. C. Hughes, J. B. Jago, I. Korovnikov, J. R. Laurie, B. S. Lieberman, J. R. Paterson, T. V. Pegel, L. E. Popov, A. W. A. Rushton, S. S. Sukhov, M. F. Tortello, Z. Zhou, A. Żylińska, in *Early Palaeozoic Biogeography and Palaeogeography,* D. A. T. Harper, T. Servais, Eds. (The Geological Society of London, 2013), pp. 273–296.

5. A. Kerner, F. Debrenne, in *Early Palaeozoic Biogeography and Palaeogeography,* D. A. T. Harper, T. Servais, Eds. (The Geological Society of London, 2013), pp. 59–66.

6. J. D. Holmes, G. E. Budd, Reassessing a cryptic history of early trilobite evolution. *Commun. Biol.* **5**, 1177 (2022).

7. G. A. Brock, M. J. Engelbretsen, J. B. Jago, P. D. Kruse, J. R. Laurie, J. H. Shergold, G. R. Shi, J. E. Sorauf, Palaeobiogeographic affinities of Australian Cambrian faunas. *Mem. Assoc. Australasian Palaeontols* **23**, 1–61 (2000).

8. J. G. Meert, B. S. Lieberman, The Neoproterozoic assembly of Gondwana and its relationship to the Ediacaran–Cambrian radiation. *Gondw. Res.* **14**, 5–21 (2008).

9. J. R. Paterson, G. D. Edgecomb, M. S. Y. Lee, Trilobite evolutionary rates constrain the duration of the Cambrian explosion. *Proc. Natl. Acad. Sci. U.S.A.* **116**, 4394–4399 (2019).

10. B. Pan, G. A. Brock, C. B. Skovsted, M. J. Betts, T. P. Topper, G. Li, *Paterimitra pyramidalis* Laurie, 1986, the first tommotiid discovered from the early Cambrian of North China. *Gondw. Res.* **63**, 179–185 (2018).

11. Z. Zhang, L. E. Popov, L. E. Holmer, Z. Zhang, Earliest ontogeny of early Cambrian acrotretoid brachiopods—First evidence for metamorphosis and its implications. *BMC Evol. Biol.* **18**, 42 (2018).

12. L. Na, A. T. Kocsis, Q. Li, W. Kiessling, Coupling of geographic range and provincialism in Cambrian marine invertebrates. *Paleobiology* **49**, 284–295 (2023).

13. D. Jablonski, K. Roy, J. W. Valentine, Out of the tropics: Evolutionary dynamics of the latitudinal diversity gradient. *Science* **314**, 102–106 (2006).

14. A. Yu. Zhuravlev, in *The Ecology of the Cambrian Radiation*, A. Yu. Zhuravlev, R. Riding, Eds. (Columbia Univ. Press, 2001), pp. 173–199.

15. R. K. Butlin, J. Galindo, J. W. Graham, Sympatric, parapatric or allopatric: The most important way to classify speciation? *Phil. Trans. R. Soc. B* **363**, 2997–3007 (2008).

16. C. E. Bird, I. Fernandez-Silva, D. J. Skillings, R. J. Toonen, Sympatric speciation in the post "Modern Synthesis" era of evolutionary biology. *Evol. Biol.* **39**, 158–180 (2012).

17. A. L. Stigall, in *Macroevolution: Explanation, Interpretation, and Evidence*, E. Serrelli, N. Gontier, Eds. (Springer, 2015), pp. 301–327.

18. E. A. Sperling, A. H. Knoll, P. R. Girguis, The ecological physiology of Earth's second oxygen revolution. *Annu. Rev. Ecol. Evol. Syst.* **46**, 215–235 (2015).

19. T. W. Dahl, J. N. Connelly, D. Li, A. Kouchinsky, B. C. Gill, S. Porter, A. C. Maloof, M. Bizzarro, Atmosphere–ocean oxygen and productivity dynamics during early animal radiations. *Proc. Natl. Acad. Sci. U.S.A.* **116**, 19352–19361 (2019).

20. T. He, M. Zhu, B. J. W. Mills, P. M. Wynn, A. Yu. Zhuravlev, R. Tostevin, P. A. E. Pogge von Strandmann, A. Yang, S. W. Poulton, G. A. Shields, Possible links between extreme oxygen perturbations and the Cambrian radiation of animals. *Nat. Geosci.* **12**, 468–474 (2019).

21. A. Yu. Zhuravlev, E. G. Mitchell, F. Bowyer, R. Wood, A. Penny, Increases in reef size, habitat and metacommunity complexity associated with Cambrian radiation oxygenation pulses. *Nat. Commun.* **13**, 7523 (2022).

22. A. Bachan, K. V. Lau, M. R. Saltzman, E. Thomas, L. R. Kump, J. L. Payne, A model for the decrease in amplitude of carbon isotope excursions across the Phanerozoic. *Am. J. Sci.* **317**, 641–676 (2017).

23. R. Tostevin, B. J. W. Mills, Reconciling proxy records and models of Earth's oxygenation during the Neoproterozoic and Palaeozoic. *Interface Focus* **10**, 20190137 (2020).

24. A. Yu. Zhuravlev, R. Wood, Dynamic and synchronous changes in metazoan body size during the Cambrian Explosion. *Sci. Rep.* **10**, 6784 (2020).

25. R. Wood, D. H. Erwin, Innovation not recovery: Dynamic redox promotes metazoan radiations. *Biol. Rev.* **93**, 863–873 (2018).

26. F. T. Bowyer, A. Yu. Zhuravlev, R. Wood, F. Zhao, S. S. Sukhov, R. D. Alexander, S. W. Poulton, M. Zhu, Implications of an integrated late Ediacaran to early Cambrian stratigraphy of the Siberian Platform, Russia. *Geol. Soc. Am. Bull.* (2023).

27. A. Yu. Zhuravlev, R. A. Wood, Anoxia as the cause of the mid-Early Cambrian (Botomian) extinction event. *Geology* **24**, 311–314 (1996).

28. M. Domeier, Early Paleozoic tectonics of Asia: Towards a full-plate model. *Geosci. Frontiers* **9**, 789–862 (2018).

29. T. H. Torsvik, L. R. M. Cocks, The integration of palaeomagnetism, the geological record and mantle tomography in the location of ancient continents. *Geol. Mag.* **156**, 242–260 (2019).

30. A. S. Merdith, S. E. Williams, A. S. Collins, M. G. Tetley, J. A. Mulder, M. L. Blades, A. Young, S. E. Armistead, J. Cannon, S. Zahirovic, R. D. Müller, Extending full-plate tectonic models into deep time: Linking the Neoproterozoic and the Phanerozoic. *Earth Sci. Rev.* **214**, 103477 (2021).

31. V. A. Astashkin, T. V. Pegel, Yu. Ya. Shabanov, S. S. Sukhov, V. M. Sundukov, L. N. Repina, A. Yu. Rozanov, A. Yu. Zhuravlev, The Cambrian System on the Siberian Platform. Correlation chart and explanatory notes. *IUGS Publ.* **27**, 1–133 (1991).

32. S. S. Sukhov, T. V. Pegel, Yu. Ya. Shabanov, *Regional Stratigraphic Chart of the Cambrian Strata of the Siberian Platform: Decisions of the All-Russian Stratigraphic Meeting on the Development of Stratigraphic Charts of the Upper Precambrian and Palaeozoic of Siberia* [in Russian] (SNIIGGiMS, 2021).

33. A. L. Yanshin, M. A. Zharkov, *Geology and Potassium-Bearing Capacities of the Cambrian Deposits in the Southwestern Part of the Siberian Platform* [in Russian] (Nauka, 1974).

34. N. V. Mel'nikov, *Vendian-Cambrian Saliniferous Basin of the Siberian Platform (Stratigraphy, Developmental History)* [in Russian] (Siberian Scientific-Research Institute of Geology, Geophysics and Mineral Resources, 2018).

35. T. Wotte, C. B. Skovsted, M. J. Whitehouse, A. Kouchinsky, Isotopic evidence for temperate oceans during the Cambrian Explosion. *Sci. Rep.* **9**, 6330 (2019).

36. T. W. W. Hearing, A. Pohl, M. Williams, Y. Donnadieu, T. H. P. Harvey, C. R. Scotese, P. Sepulchre, A. Franc, T. R. A. Vandenbroucke, Quantitative comparison of geological data and model simulations constrains early Cambrian geography and climate. *Nat. Commun.* **12**, 3868 (2021).

37. G.-Y. Wei, N. J. Planavsky, T. He, F. Zhang, R. G. Stockey, D. B. Cole, Y.-B. Lin, H.-F. Ling, Global marine redox evolution from the late Neoproterozoic to the early Paleozoic constrained by the integration of Mo and U isotope records. *Earth Sci. Rev.* **214**, 103506 (2021).

38. R. Tostevin, M. O. Clarkson, S. Gangl, G. A. Shields, R. A. Wood, F. Bowyer, A. M. Penny, C. H. Stirling, Uranium isotope evidence for an expansion of anoxia in terminal Ediacaran oceans. *Earth Planet. Sci. Lett.* **506**, 104–112 (2019).

39. M. O. Clarkson, T. C. Sweere, C. F. Chui, R. Hennekam, F. T. Bowyer, R. A. Wood, Environmental controls on very high δ^{238}U values in reducing sediments: Implications for Neoproterozoic seawater records. *Earth Sci. Rev.* **237**, 104306 (2023).

40. C. Jin, C. Li, T. J. Algeo, N. J. Planavsky, H. Cui, X. Yang, Y. Zhao, X. Zhang, S. Xie, A highly redox-heterogeneous ocean in South China during the early Cambrian (∼529–514 Ma): Implications for biota-environment co-evolution. *Earth Planet. Sci. Lett.* **441**, 38–51 (2016).

41. F. Bowyer, R. A. Wood, S. W. Poulton, Controls on the evolution of Ediacaran metazoan ecosystems: A redox perspective. *Geobiology* **15**, 516–551 (2017).

42. A. Kouchinsky, S. Bengtson, V. Pavlov, B. Runnegar, A. Val'kov, E. Young, Pre-Tommotian age of the lower Pestrotsvet Formation in the Selinde section on the Siberian platform: Carbon isotopic evidence. *Geol. Mag.* **142**, 319–325 (2005).

43. A. Yu. Rozanov, V. V. Missarzhevsky, N. A. Volkova, L. G. Voronova, I. N. Krylov, B. M. Keller, I. K. Korolyuk, K. Lendzion, R. Michniak, N. G. Pykhova, A. D. Sidorov, *The Tommotian Stage and the Cambrian Lower Boundary Problem* (English translation, Amerind Publishing Co., 1981, Nauka, 1969).

44. D. V. Osadchaya, L. N. Kashina, I. T. Zhuravleva, N. P. Borodina, A. S. Boyarinov, *Lower Cambrian Stratigraphy and Archaeocyaths of the Altay-Sayan Region* [in Russian] (Nauka, 1979).

45. F. Debrenne, A. Yu. Zhuravlev, P. D. Kruse, in *Treatise on Invertebrate Paleontology, Part E (Revised), Porifera, Volumes 4–5 (Hypercalcified Porifera)*, P. A. Selden, Ed. (University of Kansas Paleontological Institute, 2015), pp. 845–922.

46. I. T. Zhuravleva, *Archaeocyaths of the Siberian Platform* [in Russian] (USSR Academy of Sciences Publishers, 1960).

47. A. Yu. Zhuravlev, R. Wood, Lower Cambrian reefal cryptic communities. *Palaeontology* **38**, 443–470 (1995).

48. M. Klautau, A. M. Solé-Cava, R. Borojevic, Biochemical systematics of sibling sympatric species of *Clathrina* (Porifera: Calcarea). *Biochem. Syst. Ecol.* **22**, 367–375 (1994).

49. G. Wörheide, B. M. Degnan, J. N. A. Hooper, J. Reitner, in *Proceedings of the 9th International Coral Reef Symposium* (Indonesian Ministry of State for Environment, International Society for Reef Studies, 2000), vol. 1, pp. 339–346.

50. J. A. Cruz-Barraza, J. L. Carballo, A. Rocha-Olivares, H. Ehrlich, M. Hog, Integrative taxonomy and molecular phylogeny of genus *Aplysina* (Demospongiae: Verongida) from Mexican Pacific. *PLOS ONE* **7**, e42049 (2012).

51. A. Yu. Rozanov, Homological variability of archaeocyathans. *Geol. Mag.* **111**, 107–120 (1974).

52. I. T. Zhuravleva, V. I. Korshunov, A. Yu. Rozanov, in *Lower Cambrian Biostratigraphy and Palaeontology of Siberia and the Far East*, I. T. Zhuravleva, Ed. [in Russian] (Nauka, 1969), pp. 5–59.

53. A. Yu. Zhuravlev, I. T. Zhuravleva, V. D. Fonin, Archaeocyaths from the Lower Cambrian of Siberia. *Paleontol. Zh.* **1983**, 22–30 (1983).

54. A. Yu. Zhuravlev, in *Biostratigraphy and Palaeontology of the Cambrian of Northern Asia* [in Russian], L. N. Repina, Ed. (Nauka, 1990), Trans. Inst. Geol. Geophys. Siberian Branch USSR Acad. Sci., vol. 765, pp. 136–147.

55. M. D. Brasier, A. Yu. Rozanov, A. Yu. Zhuravlev, R. M. Corfield, L. A. Derry, A carbon isotope reference scale for the Lower Cambrian succession in Siberia: Report of IGCP Project 303. *Geol. Mag.* **131**, 767–783 (1994).

56. A. Kouchinsky, A. S. Bengtson, V. Pavlov, B. Runnegar, P. Torssander, E. Young, K. Ziegler, Carbon isotope stratigraphy of the Precambrian–Cambrian Sukharikha River section, northwestern Siberian platform. *Geol. Mag.* **144**, 609–618 (2007).

57. A. Kouchinsky, R. Alexander, S. Bengtson, F. Bowyer, S. Clausen, L. E. Holmer, K. A. Kolesnikov, I. V. Korovnikov, V. Pavlov, C. B. Skovsted, G. Ushatinskaya, R. Wood, A. Yu. Zhuravlev, Early–middle Cambrian stratigraphy and faunas from Northern Siberia. *Acta Palaeontol. Pol.* **67**, 341–464 (2022).

58. Yu. Ya. Shabanov, V. A. Astashkin, T. V. Pegel, L. I. Egorova, I. T. Zhuravleva, Yu. L. Pelman, V. M. Sundukov, M. V. Stepanova, S. S. Sukhov, A. B. Fedorov, B. B. Shishkin, N. V. Vaganova, V. I. Ermak, K. V. Ryabukha, A. G. Yadrenkina, G. P. Abaimova, T. V. Lopushinskaya, O. V. Sychev, T. A. Moskalenko, Lower Palaeozoic of the Southwestern Slope of the Anabar Anteclise (According to Borehole Data) [in Russian] (Nauka, 1987).

59. V. A. Datsenko, I. T. Zhuravleva, N. P. Lazarenko, Yu. N. Popov, N. E. Chernysheva, *Biostratigraphy and fauna of the Cambrian deposits of the northwestern Siberian Platform. Trans. Sci.-Res. Inst. Geol. Arctic* **155** [in Russian] (Nedra, 1968).

60. L. I. Egorova, V. E. Savistkiy, *Stratigraphy and Biofacies of the Cambrian of the Siberian Platform (Western Anabar Area). Trans. Sib. Sci.-Res. Inst. Geol. Geophys. Miner. Res.* **43** [in Russian] (Nedra, 1969).

61. L. N. Repina, N. P. Lazarenko, N. P. Meshkova, V. I. Korshunov, N. I. Nikiforov, N. A. Aksarina, *Biostratigraphy and Fauna of the Lower Cambrian of the Kharaulakh (Tuora-Sis Ridge)* [in Russian] (Nauka, 1974), Trans. Inst. Geol. Geophys. Siberian Branch USSR Acad. Sci, vol. 235.

62. I. V. Korovnikov, N. V. Novozhilova, New biostratigraphical constraints on the Lower and lower Middle Cambrian of the Kharaulakh Mountains (northeastern Siberian Platform, Chekurovka anticline). *Russ. Geol. Geophys.* **53**, 776–786 (2012).

63. A. L. Rode, B. S. Lieberman, Using GIS to unlock the interactions between biogeography, environment, and evolution in Middle and Late Devonian brachiopods and bivalves. *Palaeogeogr. Palaeoclimatol. Palaeoecol.* **211**, 345–359 (2004).

64. D. Jablonski, Extinction and the spatial dynamics of biodiversity. *Proc. Natl. Acad. Sci. U.S.A.* **105**, 11528–11535 (2008).

65. E. E. Saupe, H. Qiao, J. R. Hendricks, R. W. Portell, S. J. Hunter, J. Soberón, B. S. Lieberman, Niche breadth and geographic range size as determinants of species survival on geological time scales. *Glob. Ecol. Biogeogr.* **24**, 1159–1169 (2015).

66. W. Kiessling, M. Aberhan, Environmental determinants of marine benthic biodiversity dynamics through Triassic-Jurassic time. *Paleobiology* **33**, 414–434 (2007).

67. M. Steiner, G. Li, Y. Qian, M. Zhu, B.-D. Erdtmann, Neoproterozoic to early Cambrian small shelly fossil assemblages and a revised biostratigraphic correlation of the Yangtze Platform (China). *Palaeogeogr. Palaeoclimatol. Palaeoecol.* **254**, 67–99 (2007).

68. W. Zhao, J. Liu, R. D. C. Bicknell, Geometric morphometric assessment of Guanshan trilobites (Yunnan Province, China) reveals a limited diversity of palaeolenid taxa. *Palaeontol. Electronica* **23**, a22 (2020).

69. A. M. C. Şengör, G. Sunal, B. A. Natal'in, R. van der Voo, The Altaids: A review of twenty-five years of knowledge accumulation. *Earth Sci. Rev.* **228**, 104013 (2022).

70. T. V. Pegel, Evolution of trilobite biofacies in Cambrian basins of the Siberian Platform. *J. Paleontol.* **74**, 1000–1019 (2000).

71. V. A. Luchinina, I. V. Korovnikov, N. V. Novozhilova, D. A. Tokarev, Benthic Cambrian biofacies of the Siberian Platform (hyoliths, small shelly fossils, archeocyaths, trilobites and calcareous algae). *Stratigr. Geol. Correl.* **21**, 131–149 (2013).

72. E. N. Maksimova, K. N. Chertina, in *GeoBaikal 2018* (European Association of Geoscientists & Engineers, 2018); https://doi.org/10.3997/2214-4609.201802006.

73. S. M. Holland, M. E. Patzkowsky, Models for simulating the fossil record. *Geology* **27**, 491–494 (1999).

74. S. M. Holland, M. E. Patzkowsky, The stratigraphy of mass extinction. *Palaeontology* **58**, 903–924 (2015).

75. S. F. Bakhturov, V. M. Evtushenko, V. S. Pereladov, *Kuonamka Bituminous Carbonate-Shale Formation* (Nauka, 1988).

76. T. M. Parfenova, I. V. Korovnikov, V. G. Eder, V. N. Melenevskii, Organic geochemistry of the Lower Cambrian Sinyaya Formation (northern slope of the Aldan anteclise). *Russ. Geol. Geophys.* **58**, 586–599 (2017).

77. M. Savarese, Functional analysis of archaeocyathan skeletal morphology and its paleobiological implications. *Paleobiology* **18**, 464–480 (1992).

78. A. Yu. Zhuravlev, A functional morphological approach to the biology of the Archaeocyatha. *Neues Jahrb. Geol. Palaontol. Abh.* **190**, 315–327 (1993).

79. M. Doebeli, U. Diekmann, Speciation along environmental gradients. *Nature* **421**, 259–264 (2003).

80. O. Seehausen, C. E. Wagner, Speciation in freshwater fishes. *Annu. Rev. Ecol. Evol. Syst.* **45**, 621–651 (2014).

81. W. R. Rice, Speciation via habitat specialization: The evolution of reproductive isolation as a correlated character. *Evol. Ecol.* **1**, 301–314 (1987).

82. X. Thibert-Plante, A. P. Hendry, The consequences of phenotypic plasticity for ecological speciation. *J. Evol. Biol.* **24**, 326–342 (2011).

83. V. A. Luchinina, I. V. Korovnikov, D. P. Sipin, A. V. Fedoseev, Upper Vendian–Lower Cambrian biostratigraphy of the Sukharikha River section. *Russ. Geol. Geophys.* **38**, 1385–1397 (1997).

84. S. M. Rowland, V. A. Luchinina, I. V. Korovnikov, D. P. Sipin, A. I. Tarletskov, A. V. Fedoseev, Biostratigraphy of the Vendian-Cambrian Sukharikha River section, northwestern Siberian Platform. *Can. J. Earth Sci.* **35**, 339–352 (1998).

85. I. V. Korovnikov, V. V. Marusin, D. A. Tokarev, O. T. Obut, Trace fossils from the Vendian-Cambrian transitional strata of the Igarka Uplift (northwestern Siberian Platform). *Paleontol. J.* **53**, 566–574 (2019).

86. A. I. Goryachev, M. A. Zharkov, in *Stratigraphy of the Lower Cambrian and Upper Precambrian in the South of the Siberian Platform* [in Russian], B. S. Sokolov, Ed. (Nauka, 1969), p. 17–33.

87. M. A. Zharkov, Yu. K. Sovetov, in *Stratigraphy of the Lower Cambrian and Upper Precambrian in the South of the Siberian Platform* [in Russian], B. S. Sokolov, Ed. (Nauka, 1969), p. 34–55.

88. I. T. Zhuravleva, Yu. K. Sovetov, T. N. Titorenko, in *Stratigraphy of the Lower Cambrian and Upper Precambrian in the South of the Siberian Platform* [in Russian], B. S. Sokolov, Ed. (Nauka, 1969), p. 13–16.

89. V. V. Khomentovsky, V. Yu. Shenfil', M. S. Yakshin, S. P. Butakov, *The Reference Sections of the Upper Precambrian and Lower Cambrian Strata on the Siberian Platform*, L. N. Repina, Ed. [in Russian] (Nauka, 1972), Trans. Inst. Geol. Geophys. Siberian Branch USSR Acad. Sci., vol. 141, p. 3–32.

90. E. P. Butakov, E. G. Viks, P. P. Skorobogatykh, in *News of the Stratigraphy and Palaeontology of the Late Precambrian of the Siberian Platform* [in Russian], V. V.

Khomentovsky, Ed. (Institute of Geology and Geophysics, Siberian Branch, USSR Academy of Sciences, 1980), p. 84–104.

91. Z. A. Akul’cheva, E. M. Galperova, E. L. Drobkova, L. A. Lysova, T. N. Titorenko, A. A. Treshchetenkova, Z. Kh. Fayzulina, in *Boundary Strata of the Precambrian and Cambrian of the Siberian Platform (Biostratigraphy, Palaeontology, Conditions of the Formation)* [in Russian], N. P. Meshkova, I. V. Nikolaeva, Eds. (Nauka, 1981), pp. 65–139.

92. T. I. Burtseva, I. T. Zhuravleva, First find of archaeocyaths in the Irkutsk Amphitheatre [in Russian]. *Dokl. Akad. Nauk SSSR* **106**, 885–888 (1956).

93. V. I. Korshunov, I. T. Zhuravleva, in *New Data on the Lower Palaeozoic Biostratigraphy of the Siberian Platform* [in Russian], A. B. Ivanovskiy, B. S. Sokolov, Eds. (Nauka, 1967), p. 3–11.

94. T. G. Babkina, S. I. Ediseeva, in *Geology and Mineral Resources of the North-East of Russia: Proceedings of the All-Russian Scientific-Practical Conference, April, 2-4, 2013* [in Russian], (Institution of Advancing Training, North-Eastern Federal University, 2013), vol. 1, p. 54–57.

95. T. V. Pegel, L. I. Egorova, Yu. Ya. Shabanov, I. V. Korovnikov, V. A. Luchinina, A. K. Salikhova, V. M. Sundukov, A. B. Fedorov, A. Yu. Zhuravlev, P. Yu. Parkhaev, Yu. E. Demidenko, *Stratigraphy of Oil and Gas Basins of Siberia. Cambrian of Siberian Platform. V. 2 – Palaeontology* [in Russian] (Institute of Petroleum Geology and Geophysics, Siberian Branch, Russian Academy of Sciences, 2016).

96. V. V. Gritsik, in *Problems of Lower Cambrian Palaeontology and Biostratigraphy of Siberia and the Far East* [in Russian], I. T. Zhuravleva, Ed. (Nauka, 1969), p. 186–202.

97. S. S. Sukhov, Yu. Ya. Shabanov, T. V. Pegel, S. V. Saraev, Yu. F. Filippov, I. V. Korovnikov, V. M. Sundukov, A. B. Fedorov, A. I. Varlamov, A. S. Efimov, V. A. Kontorovich, A. E. Kontorovich, *Stratigraphy of Oil and Gas Basins of Siberia. Cambrian*

of the Siberian Platform, V. 1: Stratigraphy [in Russian] (Institute of Petroleum Geology and Geophysics, Siberian Branch, Russian Academy of Sciences, 2016).

98. B. B. Kochnev, B. G. Pokrovsky, A. B. Kuznetsov, V. V. Marusin, C and Sr isotope chemostratigraphy of Vendian-Lower Cambrian carbonate sequences in the central Siberian Platform. *Russ. Geol. Geophys.* **59**, 585–605 (2018).

99. V. A. Astashkin, B. B. Shishkin, Yu. Ya. Shabanov, V. I. Avdeeva, M. I. Alekseev, G. V. Novikov, in *Stratigraphy and Palaeontology of the Precambrian and Phanerozoic of Siberia* [in Russian], V. A. Astashkin, Ed. (Siberian Scientific-Research Institute of Geology, Geophysics and Mineral Resources, 1985), p. 22–33.

100. B. B. Shishkin, Vendian deposits of the southeastern part of Siberian Platform. *Geol. Miner. Res. Siberia* **3**, 3–10.

101. L. I. Egorova, Yu. Ya. Shabanov, A. Yu. Rozanov, V. E. Savitskiy, N. E. Chernysheva, B. B. Shishkin, *Elanka and Kuonamka Facies Stratotypes of the Lower Boundary of the Middle Cambrian in Siberia* [in Russian] (Nedra, 1976), Trans. Sib. Sci.-Res. Inst. Geol. Geophys. Miner. Res, vol. 211.

102. A. I. Varlamov, V. M. Sundukov, in *News in the Lower Palaeozoic Stratigraphy and Palaeontology of Central Siberia* [in Russian], L. N. Repina, Yu. I. Tesakov, Eds. (Institute of Geology and Geophysics, Siberian Branch, USSR Academy of Sciences, 1978), p. 27–35.

103. V. I. Korshunov, *Lower Cambrian Biostratigraphy and Archaeocyaths of the Northeastern Aldan Anteclise* [in Russian] (Yakutsk Publishers, 1972).

104. V. V. Khomentovsky, G. A. Karlova, in *Late Precambrian and Early Palaeozoic of Siberia. Siberian Platform and Its Borderland* [in Russian], V. V. Khomentovsky (United Institute of Geology, Geophysics and Mineralogy, Siberian Branch, USSR Academy of Sciences, 1991), p. 3–44.

105. V. V. Khomentovsky, A. K. Val'kov, G. A. Karlova, S. V. Nuzhnov, in *Late Precambrian and Early Palaeozoic of Siberia. Vendian Strata* [in Russian], V. V. Khomentovsky, Ed.

(Institute of Geology and Geophysics, Siberian Branch, USSR Academy of Sciences, 1983), p. 24–36.

106. V. V. Khomentovsky, A. K. Val'kov, G. A. Karlova, in *Late Precambrian and Early Palaeozoic of Siberia. Problems of the Regional Stratigraphy* [in Russian], V. V. Khomentovsky, A. S. Gibsher, Eds (Institute of Geology and Geophysics, Siberian Branch, USSR Academy of Sciences, 1990) p. 3–57.

107. V. I. Korshunov, L. N. Repina, V. A. Sysoev, To the structure of the Pestrotsvet Formation on the East of the Aldan Anteclise [in Russian]. *Geol. Geofiz.* **1969**, 18–21 (**1969**).

108. L. N. Repina, I. T. Zhuravleva, in *Environment and Life in the Geological Past (Facies and Organisms)* [in Russian], O. A. Betekhtina, I. T. Zhuravleva, Eds. (Nauka, 1977), p. 134–136.

109. L. N. Repina, Z. V. Borodaevskaya, V. V. Ermak, in *Cambrian of Siberia and Middle Asia*, I. T. Zhuravleva, L. N. Repina, Eds. [in Russian] (Moscow, Nauka, 1988), Trans. Inst. Geol. Geophys. Siberian Branch USSR Acad. Sci., vol. 720, p. 3–31.

110. V. V. Khomentovsky, G. A. Karlova, The boundary between Nemakit-Daldynian and Tommotian stages (Vendian-Cambrian Systems) of Siberia. *Stratigr. Geol. Correl.* **10**, 217–239 (2002).

111. V. V. Khomentovsky, G. A. Karlova, in *Late Precambrian and Early Palaeozoic of Siberia. Actual Problems of the Stratigraphy* [in Russian], V. V. Khomentovsky, Yu. K. Sovetov (Institute of Geology and Geophysics, Siberian Branch, USSR Academy of Sciences, 1989), p. 23–61.

112. Yu. L. Pelman, V. V. Ermak, A. B. Fedorov, V. A. Luchinina, I. T. Zhuravleva, L. N. Repina, V. I. Bondarev, Z. V. Borodaevskaya, in *Biostratigraphy and Palaeontology of the Cambrian of Northern Asia* [in Russian], L. N. Repina, Ed. (Nauka, 1990), Trans. Inst. Geol. Geophys. Siberian Branch USSR Acad. Sci., vol. 765, p. 3–32.

113. A. I. Varlamov, N. V. Grigor'eva, A. Yu. Zhuravlev, I. T. Zhuravleva, L. N. Repina, A. Yu. Rozanov, Yu. Ya. Shabanov, in *Early Cambrian Stage Subdivision. Stratigraphy* [in Russian], A. Yu. Rozanov, B. S. Sokolov, Eds. (Nauka, 1984), p. 20–93.

114. V. M. Sundukov, in *New Species of Ancient Plants and Invertebrates from the Phanerozoic of Siberia* [in Russian], S. P. Bulynnikova, I. G. Klimova, Eds. (Siberian Scientific-Research Institute of Geology, Geophysics and Mineral Resources, 1987), p. 46–51.

115. V. M. Sundukov, in *Stratigraphy and Facies of Sedimentary Basins of Siberia* [in Russian], V. A. Astashkin, Ed. (Siberian Scientific-Research Institute of Geology, Geophysics and Mineral Resources, 1982), p. 46–56.

116. A. Yu. Rozanov, in *Problems of Lower Cambrian Palaeontology and Biostratigraphy of Siberia and the Far East* [in Russian], I. T. Zhuravleva, Ed. (Nauka, 1969), p. 106–113.

117. I. V. Korovnikov, Trilobites of the suborder Eodiscina from the Lower Cambrian of the northeastern Siberian Platform (Khorbosuonka River section). *Paleontol. J.* **41**, 614–620 (2007).

118. I. N. Dyatlova, A. N. Donov, V. R. Trofimov, in *Biostratigraphy and Palaeontology of the Cambrian of Northern Asia*, L. N. Repina, Ed. [in Russian] (Nauka, 1990), Trans. Inst. Geol. Geophys. Siberian Branch USSR Acad. Sci. vol. 765, p. 123–135.

119. V. I. Bondarev, M. A. Minaeva, in *Cambrian of Siberia and the Middle East* [in Russian], I. T. Zhuravleva, L. N. Repina, Eds. (Nauka, 1988), Trans. Inst. Geol. Geophys. Siberian Branch USSR Acad. Sci. vol. 720, p. 97–110.

120. A. Yu. Rozanov, Yu. M. Fomin, in *Problems of Lower Cambrian Palaeontology and Biostratigraphy of Siberia* [in Russian], I. T. Zhuravleva, Ed. (Nauka, 1972), p. 233–234.

121. V. M. Sundukov, A. B. Fedorov, in *Biostratigraphy and Palaeontology of the Cambrian of Northern Asia* [in Russian], I. T. Zhuravleva, Ed. (Nauka, 1986), Trans. Inst. Geol. Geophys. Siberian Branch USSR Acad. Sci., vol. 669, p. 108–119.

122. I. T. Zhuravleva, in *Biostratigraphy and Palaeontology of the Lower and Middle Cambrian of Northern Asia* [in Russian], N. P. Meshkova Ed. (Nauka, 1983), Trans. Inst. Geol. Geophys. Siberian Branch USSR Acad. Sci., vol. 541, p. 81–94.

123. V. M. Sundukov, New archaeocyaths from the Lower Cambrian of the Lena and Kotuy. *Paleontol. Zh.* **1983**, 13–17 (1983).

124. A. Yu. Zhuravlev, E. B. Naimark, R. A. Wood, Controls on the diversity and structure of earliest metazoan communities: Early Cambrian reefs from Siberia. *Earth Sci. Rev.* **147**, 18–29 (2015).

125. M. Zhu, A. Yu. Zhuravlev, R. A. Wood, F. Zhao, S. S. Sukhov, A deep root for the Cambrian explosion: Implications of new bio- and chemostratigraphy from the Siberian Platform. *Geology* **45**, 459–462 (2017).

126. M. A. Semikhatov, A. B. Kuznetsov, V. N. Podkovyrov, J. K. Bartley, Yu. V. Davydov, The Yudoma Group of Stratotype Area: C-isotope Chemostratigraphic Correlations and Yudomian-Vendian Relation. *Stratigr. Geol. Correl.* **12**, 435–459 (2004).

127. M. D. Brasier, V. V. Khomentovsky, R. M. Corfield, Stable isotopic calibration of the earliest skeletal fossil assemblages in eastern Siberia (Precambrian-Cambrian boundary). *Terra Nova* **5**, 225–232 (1993).

128. M. Magaritz, ^{13}C minima follow extinction events: A clue to faunal radiation. *Geology* **17**, 337–340 (1989).

129. M. Magaritz, W. T. Holser, J. L. Kirschvink, Carbon-isotope events across the Precambrian/Cambrian boundary on the Siberian Platform. *Nature* **320**, 258–259 (1986).

130. S. M. Pelechaty, A. J. Kaufman, J. P. Grotzinger, Evaluation of δ^{13}C chemostratigraphy for intrabasinal correlation: Vendian strata of northeast Siberia. *Geol. Soc. Am. Bull.* **108**, 992–1003 (1996).

131. B. G. Pokrovsky, M. I. Bujakaite, O. V. Kokin, Geochemistry of C, O, and Sr Isotopes and Chemostratigraphy of Neoproterozoic Rocks in the Northern Yenisei Ridge. *Lithol. Miner. Resour.* **47**, 177–199 (2012).

132. A. C. Maloof, S. M. Porter, J. L. Moore, F. Ö. Dudás, S. A. Bowring, J. A. Higgins, D. A. Fike, M. P. Eddy, The earliest Cambrian record of animals and ocean geochemical change. *Geol. Soc. Am. Bull.* **122**, 1731–1774 (2010).

133. A. J. Kaufman, A. H. Knoll, M. A. Semikhatov, J. P. Grotzinger, S. B. Jacobsen, W. Adams, Integrated chronostratigraphy of Proterozoic-Cambrian boundary beds in the western Anabar region, northern Siberia. *Geol. Mag.* **133**, 509–533 (1996).

134. A. Kouchinsky, S. Bengtson, V. V. Missarzhevsky, S. Pelechaty, P. Torssander, A. K. Val'kov, Carbon isotope stratigraphy and the problem of a pre-Tommotian Stage in Siberia. *Geol. Mag.* **138**, 387–396 (2001).

135. A. Kouchinsky, S. Bengtson, E. Landing, M. Steiner, M. Vendrasco, K. Ziegler, Terreneuvian stratigraphy and faunas from the Anabar Uplift, Siberia. *Acta Palaeontol. Pol.* **62**, 311–440 (2017).

136. I. A. Vishnevskaya, E. F. Letnikova, N. I. Vetrova, B. B. Kochnev, S. I. Dil, Chemostratigraphy and detrital zircon geochronology of the Neoproterozoic Khorbusuonka Group, Olenek Uplift, Northeastern Siberian platform. *Gondwana Res.* **51**, 255–271 (2017).

137. A. H. Knoll, J. P. Grotzinger, A. J. Kaufman, P. Kolosov, Integrated approaches to terminal Proterozoic stratigraphy: An example from the Olenek Uplift, northeastern Siberia. *Precambrian Res.* **73**, 251–270 (1995).

138. H. Cui, D. V. Grazhdankin, S. Xiao, S. Peek, V. I. Rogov, N. V. Bykova, N. E. Sievers, X. M. Liu, A. J. Kaufman, Redox-dependent distribution of early macro-organisms: Evidence from the terminal Ediacaran Khatyspyt Formation in Arctic Siberia. *Palaeogeogr. Palaeoclimatol. Palaeoecol.* **461**, 122–139 (2016).

139. M. Magaritz, J. L. Kirschvink, A. J. Latham, A. Yu. Zhuravlev, A. Yu. Rozanov, Precambrian/Cambrian boundary problem: Carbon isotope correlations for Vendian and Tommotian time between Siberia and Morocco. *Geology* **19**, 847–850 (1991).

140. J. L. Kirschvink, M. Magaritz, R. L. Ripperdan, A. Yu. Zhuravlev, A. Yu. Rozanov, The Precambrian-Cambrian Boundary: Magnetostratigraphy and Carbon Isotopes Resolve Correlation Problems Between Siberia, Morocco, and South China. *GSA Today* **1**, 69–91 (1991).

141. A. B. Kuznetsov, G. V. Ovchinnikova, I. M. Gorokhov, E. F. Letnikova, O. K. Kaurova, G. V. Konstantinova, Age constraints on the Neoproterozoic Baikal Group from combined Sr isotopes and Pb-Pb dating of carbonates from the Baikal type section, southeastern Siberia. *J. Asian Earth Sci.* **62**, 51–66 (2013).

142. S. M. Pelechaty, Integrated chronostratigraphy of the Vendian System of Siberia: Implications for a global stratigraphy. *J. Geol. Soc.* **155**, 957–973 (1998).

143. B. G. Pokrovsky, V. A. Melezhik, M. I. Bujakaite, Carbon, oxygen, Strontium, and Sulfur Isotopic Compositions in Late Precambrian Rocks of the Patom Complex, Central Siberia: Communication 1. Results, Isotope Stratigraphy, and Dating Problems. *Lithol. Miner. Resour.* **41**, 450–474 (2006).

144. J. K. Bartley, M. Pope, A. H. Knoll, M. A. Semikhatov, P. Yu. Petrov, A Vendian-Cambrian boundary succession from the northwestern margin of the Siberian Platform: Stratigraphy, palaeontology, chemostratigraphy and correlation. *Geol. Mag.* **135**, 473–494 (1998).

145. V. V. Marusin, B. B. Kochnev, G. A. Karlova, K. E. Nagovitsin, Resolving Terreneuvian stratigraphy in subtidal–intertidal carbonates: Palaeontological and chemostratigraphical evidence from the Turukhansk Uplift, Siberian Platform. *Lethaia* **52**, 464–485 (2019).

146. B. G. Pokrovsky, M. I. Bujakaite, O. L. Petrov, A. A. Kolesnikova, The C, O, and Sr Isotope Chemostratigraphy of the Vendian (Ediacaran)-Cambrian Transition, Olekma River, Western Slope of the Aldan Shield. *Stratigr. Geol. Correl.* **28**, 479–492 (2020).

147. V. V. Marusin, A. A. Kolesnikova, B. B. Kochnev, N. B. Kuznetsov, B. G. Pokrovsky, T. V. Romanyuk, G. A. Karlova, S. V. Rud'ko, A. V. Shatsillo, A. S. Dubenskiy, V. S. Sheshukov, S. M. Lyapunov, Detrital zircon age and biostratigraphic and chemostratigraphic constraints on the Ediacaran-Cambrian transitional interval in the Irkutsk Cis-Sayans Uplift, southwestern Siberian Platform. *Geol. Mag.* **158**, 1156–1172 (2021).

148. Yu. Ya. Shabanov, I. V. Korovnikov, V. S. Pereladov, K. L. Pak, A. F. Fefelov, The section of the Kuonamka Formation of the Molodo River- a candidate for a global stratigraphy of the lower boundary of the Middle Cambrian (East Siberian Platform). In *Cambrian sections of the Siberian Platform – stratotype candidates for an international stratigraphic scaling (Stratigraphy and Palaeontology). Material of the 13th international field conference of the Cambrian subdivision working group, Yakutia*, 60–70 (2008).
